# Synthesis of Analogues of Gingerol and Shogaol, the Active Pungent Principles from the Rhizomes of *Zingiber officinale* and Evaluation of Their Anti-Platelet Aggregation Effects

**DOI:** 10.3390/ijms15033926

**Published:** 2014-03-04

**Authors:** Hung-Cheng Shih, Ching-Yuh Chern, Ping-Chung Kuo, You-Cheng Wu, Yu-Yi Chan, Yu-Ren Liao, Che-Ming Teng, Tian-Shung Wu

**Affiliations:** 1 Department of Chemistry, National Cheng Kung University, Tainan 701, Taiwan; E-Mails: l3894106@mail.ncku.edu.tw (H.-C.S.); hope.wu@ge.com (Y.-C.W.); truthloveroy@yahoo.com.tw (Y.-R.L.); 2 Department of Applied Chemistry, National Chiayi University, Chiayi 600, Taiwan; E-Mail: cychern@mail.ncyu.edu.tw; 3 Department of Biotechnology, National Formosa University, Yunlin 632, Taiwan; E-Mail: pcckuoo@nfu.edu.tw; 4 Department of Biotechnology, Southern Taiwan University, Tainan 710, Taiwan; E-Mail: yuyichan@mail.stust.edu.tw; 5 College of Medicine, Pharmacological Institute, National Taiwan University, Taipei 100, Taiwan; E-Mail: cmteng@ntu.edu.tw

**Keywords:** *Zingiber officinale*, ginger, gingerol, shogaol, anti-platelet aggregation

## Abstract

The present study was aimed at discovering novel biologically active compounds based on the skeletons of gingerol and shogaol, the pungent principles from the rhizomes of *Zingiber officinale*. Therefore, eight groups of analogues were synthesized and examined for their inhibitory activities of platelet aggregation induced by arachidonic acid, collagen, platelet activating factor, and thrombin. Among the tested compounds, [6]-paradol (5b) exhibited the most significant anti-platelet aggregation activity. It was the most potent candidate, which could be used in further investigation to explore new drug leads.

## 1. Introduction

**Figure 1.**
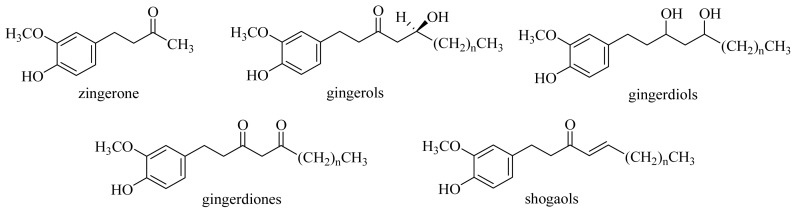
Chemical structures of the pungent principles from the rhizomes of *Zingiber officinale*.

Ginger (Chinese name: Shengjiang), derived from the rhizomes of *Zingiber officinale* Roscoe, is a well-known spice and is most frequently prescribed as a traditional Chinese medicine for its stomachic, antiemetic, antidiarrheal, expectorant, antiasthmatic, hemostatic and cardiologic properties for the treatment of several gastrointestinal and respiratory diseases [1–3]. The most famous traditional medicinal application of *Z. officinale* is to promote blood circulation for the removal of blood stasis, a mechanism that is related to anti-platelet aggregation activity [4,5]. Numerous chemical investigations of the pungent and bioactive principles of ginger have been carried out [6–19]. The pungent principles reported from the rhizomes of *Zingiber officinale* include: zingerone, gingerols, gingerdiols, gingerdiones, and shogaols (Figure 1).

**Figure 2.**
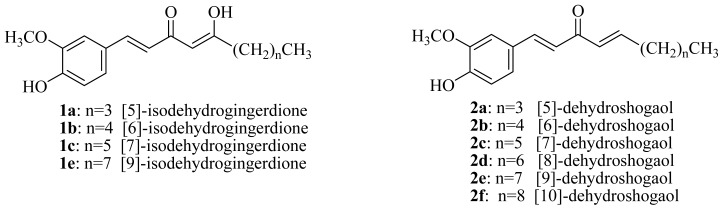

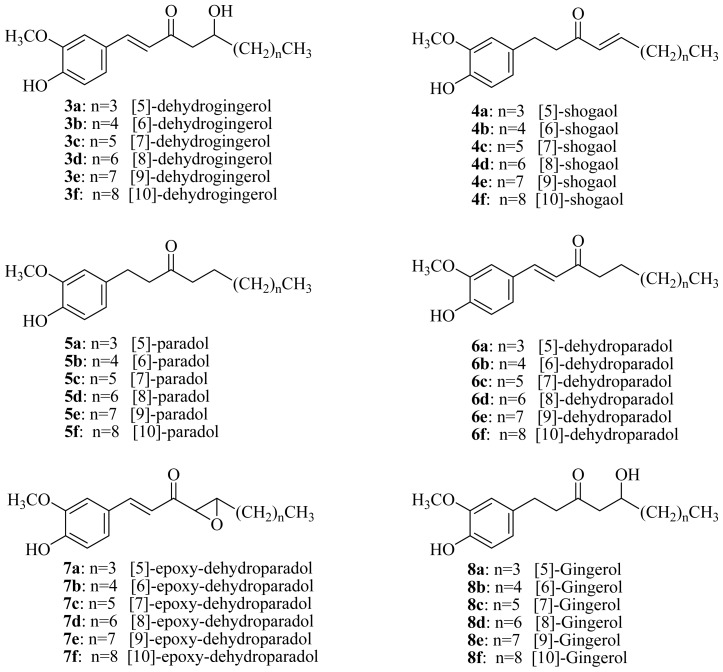
Chemical structures of the synthetic analogues of gingerol and shogaol.

In the course of our continuing research program aimed at discovering novel bioactive constituents from natural sources, thrombolytic and vasoactive activity examinations were carried out, and the ether extracts of the rhizomes of *Z. officinale* were found to exhibit significant anti-platelet aggregation activity and vasorelaxing effects. In our previous article [20], twenty-nine compounds were identified, and [6]-gingerol and [6]-shogaol exhibited potent anti-platelet aggregation bioactivity. These results initiated our interest in searching for more potent antiplatelet aggregation agents from the analogues of gingerol and shogaol. Therefore, in the present study eight groups of compounds (Figure 2) were prepared and subjected to examinations of their anti-platelet aggregation activity.

## 2. Results and Discussion

### 2.1. Chemistry

At first, the dehydrozingerone **9** was prepared by vanillin condensation with a good yield (89%), Equation (1). Then the cross aldol condensations of α,β-unsaturated ketone **9** with different aldehydes were investigated using various bases as catalysts. The major products were the dehydrogingerols **3a**–**f**, and the minor products dehydroshogaols **2a**–**f** were obtained in the optimum yield (6%–15%) when lithium bis(trimethylsilyl)amide (LiHMDS) was employed. Therefore, deprotonation of **9** with LiHMDS in tetrahydrofuran at 0 °C and subsequent trapping with aldehydes Equation (2) afforded products **2a**–**f** and **3a**–**f** with moderate yields in a range between 50% and 66% (Table 1).

(1)
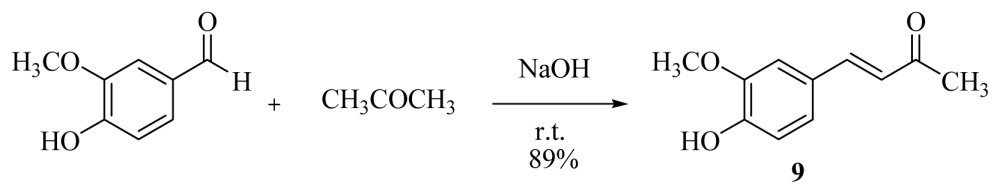


(2)
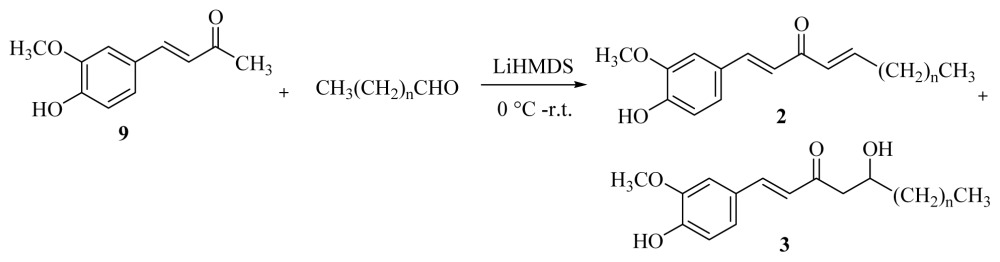


However, similar reaction conditions under air atmosphere furnished low yields of [*n*]-epoxy-dehydroparadols **7a**–**f** (Equation (3), Table 2), and comparatively, relatively higher yields of dehydroshogaols **2a**–**f** (15%–21%).

(3)
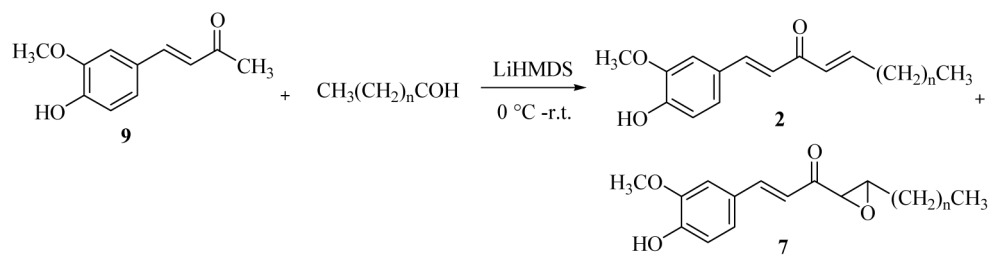


Chlorination and dehydrohalogenation of alcohols **3a**–**f** with HCl and K_2_CO_3_, respectively, produced quantitative yields of adducts **2a**–**f** Equation (4) and reduced the occurrence of trace amounts of **10**. The catalytic hydrogenation of [*n*]-dehydroshogaols **2a**–**f** over palladium on charcoal afforded [*n*]-paradols **5** and trace amounts of secondary alcohol **11**. It was surprising that [*n*]-dehydroparadols **6** could be obtained with the same method only reduced the amount of palladium on charcoal from 0.05 to 0.015 eq. The results are shown in Equation (5) and Table 3.

(4)
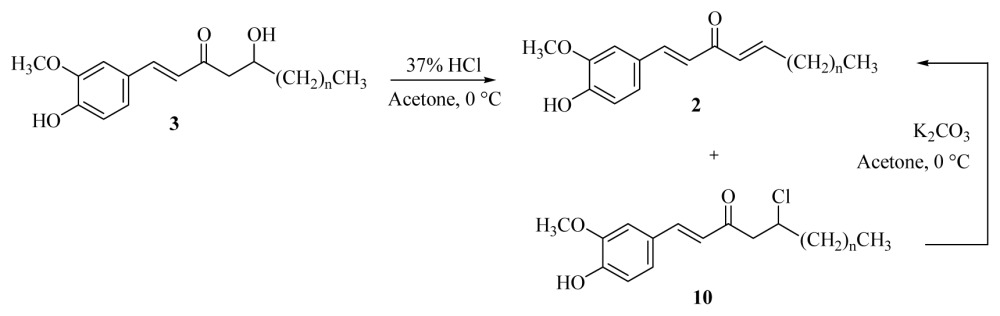


(5)
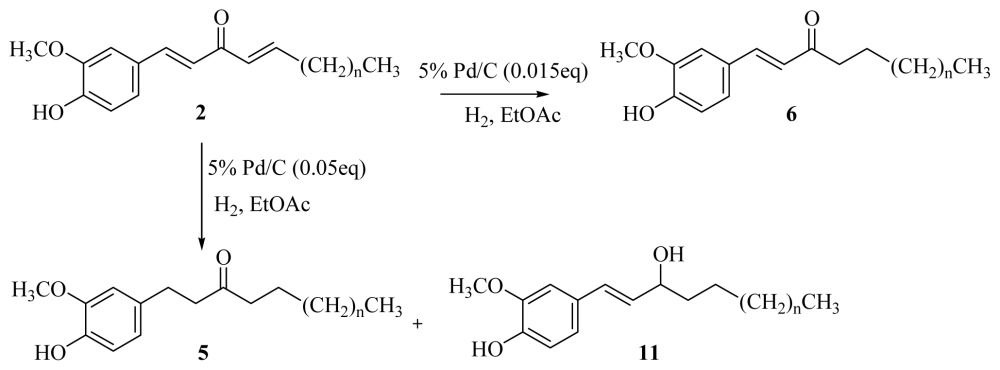


(6)
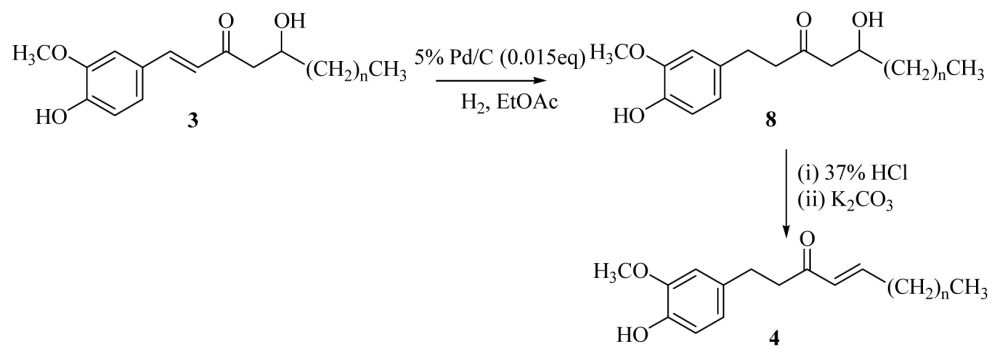


The same hydrogenation procedure was applied in [*n*]-dehydrogingerols **3**, and a high yield of [*n*]-gingerols **8** was obtained (83%–86%). Dehydration of **8** with HCl/K_2_CO_3_ gave approximately 85% of [*n*]-shogaols **4** (Equation (6), Table 4). Although there were many reagents available for the oxidation of secondary alcohols to ketone, unfortunately, most of these oxidizing agents did not show sufficient activity except in the case of Swern oxidation, which yielded a moderate amount of oxidized compound **1** (Equation (7), Table 5).

(7)
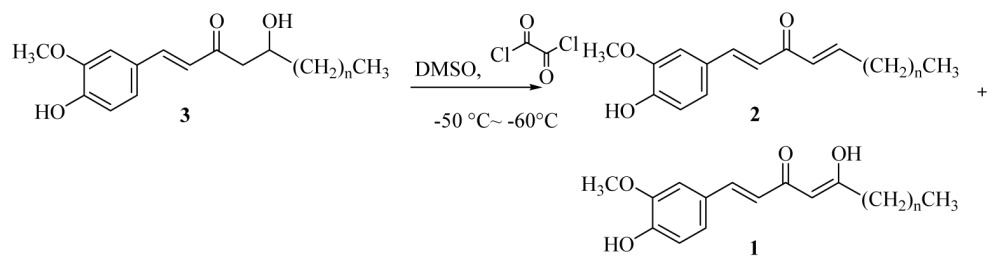


### 2.2. Anti-Platelet Aggregation Evaluation Bioassay

Platelets circulate in the blood of mammals and are involved in hemostasis, leading to the formation of blood clots. Too many platelets form blood clots that may obstruct blood vessels and induce strokes, myocardial infarctions, and pulmonary embolisms. Sometimes this situation also results in the blockage of blood vessels to other parts of the body, including the extremities of the arms or legs [21]. The traditional medicinal use of ginger is to promote the blood circulation necessary for removing blood stasis. Therefore, synthetic derivatives were examined in the anti-platelet aggregation bioassay to test for the presence of activity. The anti-platelet aggregation results are summarized in Tables 6–13. All the tested compounds displayed significant inhibitory effects on the aggregation of washed rabbit platelets stimulated by arachidonic acid (AA). At a 10 μg/mL concentration, most of the tested compounds with the exception of **3a**, **3d**, and **7e** caused the inhibition percentages of aggregation induced by AA (100 μM) to be higher than 90%. On the other hand, the activities of these synthetic derivatives against platelet activating factor (PAF) and thrombin (Thr) induced aggregation were insignificant.

Among these derivatives, the [*n*]-paradols (**5a**–**f**) series were the most active compounds, and [6]-paradol **5b** displayed the most significant inhibition, with an *IC*_50_ value of 70 ng/mL (Table 6). [*n*]-Dehydroparadols (**6a**–**f**) were generally less potent than the corresponding [*n*]-paradols (**5a**–**f**) derivatives (Table 7). The most potent compound was [10]-dehydroparadol **6f** (*n =* 8), with an *IC*_50_ value of 160 ng/mL, which was a 2.3-fold decrease in activity due to the introduction of an unsaturated C=C bond. The [*n*]-shogaols (**4a**–**f**) series (Table 8) also displayed weaker inhibition of aggregation induced by AA (100 μM) compared to their related [*n*]-paradol derivatives **5a**–**f**; however, they were more active than the [*n*]-dehydroparadols (**6a**–**f**) series. It was evident that introduction of unsaturated C=C bonds would decrease the inhibitory activity. But the location of unsaturated C=C bonds also influences the inhibitory activity. The [*n*]-dehydroshogaols (**2a**–**f**) (Table 9) which possess one more α,β-unsaturation C=C bond exhibited further decreased inhibitory activity. However, their inhibition aggregation potency induced by collagen (Col) was generally more significant than their [*n*]-paradol counterparts **5a**–**f**. Among the [*n*]-shogaols (**4a**–**f**) and [*n*]-dehydroshogaols (**2a**–**f**), [10]-shogaols (**4f**) (*n =* 8) exhibited the most significant inhibition of aggregation induced by Col (10 μM), with an *IC*_50_ value lower than 5 μg/mL. The possible mechanism was one in which the rigid styryl carbonyl ethylene prevented the alkyl tail from turning sideways, where a putative hydrophobic pocket may have been located. Therefore, a free alkyl chain could overcome such an effect.

The addition of a β-hydroxyl group to the α,β-unsaturated ketone to afford [*n*]-gingerols **8a**–**f**, resulted in a reduction of anti-platelet aggregation activity (Table 10). The introduction of an unsaturated C=C bond to the gingerol skeleton as described above (**3a**–**f**) also reduced the inhibition percentages (Table 11). Similarly, a longer side chain produced a more potent derivative. Therefore, both [10]-gingerol **8f** and [10]-dehydrogingerol **3f** displayed more significant inhibition of aggregation induced by AA (100 μM) as compared to the analogues with shorter side chains.

The [*n*]-isodehydrogingerdiones **1a**–**e** also showed significant inhibition of platelet aggregation induced by AA (Table 12). [7]-Isodehydrogingerdione **1c** was found to be the most effective compound among this series, with an *IC*_50_ value of 0.68 μg/mL. Moreover, an epoxide ring next to the α,β-unsaturated ketone produced derivatives **7a**–**f** of lower potency compared with [*n*]-paradols **5a**–**f**. They were only as potent as the dehydroshogaol series, with *IC*_50_ values between 0.96 and 2.38 μg/mL. Apparently, [10]-epoxydehydroparadol **7f** exhibited the most significant inhibitory effect among this series with an *IC*_50_ value of 0.96 μg/mL (Table 13).

## 3. Experimental Section

### 3.1. General

All the chemicals were purchased from Merck KGaA (Darmstadt, Germany), unless specifically indicated. Column chromatography was performed on silica gel (70–230 mesh, 230–400 mesh), and TLC monitoring was executed on Merck precoated Si gel 60 F_254_ plates, using UV light to visualize the spots. The melting points of the purified compounds were determined using a Yanagimoto micromelting point measuring apparatus (Tokyo, Japan) without corrections. The UV spectra were obtained on a Hitachi UV-3210 spectrophotometer (Tokyo, Japan). The IR spectra were obtained as KBr discs on a Jasco Report-100 FT-IR spectrometer (Tokyo, Japan). ^1^H and ^13^C NMR spectra were recorded on a Bruker AC-200 NMR spectrometer (Bruker, Billerica, MA, USA). Chemical shifts are shown in δ values (ppm) with tetramethylsilane as an internal standard. The EI mass and high-resolution mass spectra were measured on a VG Analytical Model 70-250S spectrometer (Micromass, Manchester, UK). Elemental analyses were performed on a Perkin-Elmer 240 analyzer (Waltham, MA, USA).

### 3.2. Synthesis of Derivatives and Spectral Data

#### 3.2.1. Preparation of Dehydrozingerone (**9**)

10% Sodium hydroxide (7.0 g, 175 mmol) was added dropwise to a solution of vanillin (2.5 g, 16.4 mmol) in acetone (100 mL) at room temperature. The reaction mixture was stirred for 12 h, concentrated under reduced pressure, then neutralized by cold 5% HCl_(aq)_. The solution was extracted with EtOAc (4 × 50 mL). The organic layers were combined, washed with saturated NaCl_(aq)_ (brine), dried over MgSO_4_, and concentrated under reduced pressure. The product was isolated on silica gel column chromatography (EtOAc/hexanes = 1/4) to afford yellow needles (2.8 g, 89% yield).

**Dehydrozingerone** (**9**): mp 126–127 °C (lit. 128–129 °C) [1]; UV (MeOH) λ_max_ 337, 299 (sh), 249 nm; IR (KBr) ν_max_ 3312, 2949, 2848, 1670, 1639, 1581, 1515, 1427, 1298, 1218, 1024, 829 cm^−1; 1^H-NMR (CDCl_3_) δ 7.44 (1H, d, *J =* 16.0 Hz, H-1), 7.09 (1H, dd, *J =* 8.2, 1.8 Hz, H-6′), 7.05 (1H, d, *J =* 1.8 Hz, H-2′), 6.92 (1H, d, *J =* 8.0 Hz, H-5′), 6.58 (1H, d, *J =* 16.0 Hz, H-2), 6.02 (1H, br s, -OH), 3.93 (3H, s, -OCH_3_), 2.36 (3H, s, H-4); ^13^C-NMR (CDCl_3_) δ 198.4, 148.2, 146.7, 143.7, 126.8, 124.9, 123.4, 114.7, 109.2, 55.9, 27.2; EIMS *m*/*z* (*rel. int*.) 192 (M^+^, 93), 190 (20), 177 (100), 145 (47), 134 (21), 117 (27), 89 (31), 78 (23), 77 (24), 51 (24).

#### 3.2.2. General Procedure for the Synthesis of [*n*]-Dehydroshogaols (**2a**–**f**) and [*n*]-Dehydrogingerols (**3a**–**f**)

A 1.0 M THF solution of lithium bis(trimethylsilyl)amide (20.8 mL) was added dropwise to a solution of dehydrozingerone (**9**) (2.0 g, 10.4 mmol) in dry THF (10 mL) at 0 °C under argon. After the mixture had been stirred for 1 h, the appropriate aldehyde (10.5 mmol) was added and stirred for another 15 min. The reaction was then quenched with 5% HCl_(aq)_ at 0 °C and extracted with EtOAc (4 × 20 mL). The organic layers were combined, washed with brine, dried over MgSO_4_, and concentrated under reduced pressure. Products **2** and **3** were isolated using silica gel column chromatography (EtOAc/CH_2_Cl_2_ = 1/16).

**[5]-Dehydroshogaol (2a)**: yellow syrup (9%); UV (MeOH) λ_max_ (log ɛ) 356 (4.14), 255 (4.03) nm; IR (neat) ν_max_ 3325, 2958, 2860, 1652, 1625, 1579, 1514, 1460, 1276, 1126, 1031 cm^−1; 1^H-NMR (CDCl_3_) δ 7.57 (1H, d, *J =* 15.8 Hz, H-1), 7.13 (1H, dd, *J =* 8.2, 2.0 Hz, H-6′), 7.06 (1H, d, *J =* 2.0 Hz, H-2′), 6.99 (1H, dt, *J =* 15.6, 6.8 Hz, H-5), 6.92 (1H, d, *J =* 8.2 Hz, H-5′), 6.80 (1H, d, *J =* 15.8 Hz, H-2), 6.44 (1H, dt, *J =* 15.6, 1.4 Hz, H-4), 6.04 (1H, br s, -OH), 3.93 (3H, s, -OCH_3_), 2.28 (2H, tdd, *J =* 6.8, 6.8, 1.4 Hz, H-6), 1.57–1.26 (4H, m, H-7, -8), 0.92 (3H, t, *J =* 7.0 Hz, H-9); ^13^C-NMR (CDCl_3_) δ 189.3, 148.2, 148.0, 146.8, 143.3, 129.0, 127.4, 123.3, 122.8, 114.8, 109.7, 56.0, 32.4, 30.3, 22.3, 13.8; EIMS *m*/*z* (*rel. int*.) 260 (M^+^, 66), 259 (26), 217 (56), 177 (80), 168 (45), 152 (65), 151 (100), 137 (40), 123 (21), 111 (35), 97 (35), 91 (23), 71 (44), 69 (55), 57 (94), 55 (85); HREIMS *m*/*z* 260.1410 [M]^+^ (Calcd for C_16_H_20_O_3_, 260.1412).

**[5]-Dehydrogingerol (3a)**: yellow needles (65%), mp 143–144 °C (lit. 144–146 °C) [22]; UV (MeOH) λ_max_ (log ɛ) 340 (4.46), 271 (sh) (3.62), 244 (4.11) nm; IR (KBr) ν_max_ 3341, 3150, 2926, 2855, 1626, 1583, 1514, 1284, 1031, 972, 816 cm^−1; 1^H-NMR (CDCl_3_) δ 7.50 (1H, d, *J =* 16.2 Hz, H-1), 7.11 (1H, dd, *J =* 8.0, 1.8 Hz, H-6′), 7.05 (1H, d, *J =* 1.8 Hz, H-2′), 6.93 (1H, d, *J =* 8.0 Hz, H-5′), 6.58 (1H, d, *J =* 16.2 Hz, H-2), 6.02 (1H, br s, -OH), 4.13(1H, m, H-5), 3.93 (3H, s, -OCH_3_), 2.88 (1H, dd, *J =* 17.0, 2.8 Hz, H-4), 2.73 (1H, dd, *J =* 17.0, 8.8 Hz, H-4), 1.58–1.35 (6H, m, H-6~8), 0.92 (3H, t, *J =* 6.6 Hz, H-9); ^13^C-NMR (CDCl_3_) δ 200.9, 148.5, 146.8, 143.8, 126.6, 124.1, 123.7, 114.8, 109.4, 67.9, 56.0, 46.4, 36.2, 27.7, 22.6, 14.0; EIMS *m*/*z* (*rel. int*.) 278 (M^+^, 33), 192 (37), 177 (100), 150 (38), 145 (37), 137 (38), 89 (14); Anal. Calcd for C_16_H_20_O_4_: C, 69.06%; H, 7.91%; Found: C, 69.09%; H, 7.85%.

**[6]-Dehydroshogaol (2b)**: yellow syrup (15%); UV (MeOH) λ_max_ (log ɛ) 355 (4.02), 258 (3.93) nm; IR(neat) ν_max_ 3354, 2956, 2856, 1654, 1625, 1581, 1514, 1267, 1207, 1033 cm^−1; 1^H-NMR (CDCl_3_) δ 7.58 (1H, d, *J =* 15.8 Hz, H-1), 7.14 (1H, dd, *J =* 8.2, 1.8 Hz, H-6′), 7.07 (1H, d, *J =* 1.8 Hz, H-2′), 7.00 (1H, dt, *J =* 15.6, 7.0 Hz, H-5), 6.94 (1H, d, *J =* 8.2 Hz, H-5′), 6.81 (1H, d, *J =* 15.8 Hz, H-2), 6.43 (1H, dt, *J =* 15.6, 1.4 Hz, H-4), 5.97 (1H, br s, -OH), 3.94 (3H, s, -OCH_3_), 2.27 (2H, tdd, *J =* 7.0, 6.8, 1.4 Hz, H-6), 1.57–1.25 (6H, m, H-7~9), 0.90 (3H, t, *J =* 6.7 Hz, H-10); ^13^C-NMR (CDCl_3_) δ 189.3, 148.1, 148.0, 147.2, 143.3, 129.0, 127.4, 123.3, 122.8, 114.8, 109.7, 56.0, 32.7, 31.4, 27.9, 22.4, 14.0; EIMS *m*/*z* (*rel. int*.) 274 (M^+^, 100), 273 (36), 217 (82), 177 (81), 152 (21), 151 (36), 145 (20), 137 (45), 57 (36), 55 (44); HREIMS *m*/*z* 274.1571 [M]^+^ (Calcd for C_17_H_22_O_3_, 274.1568).

**[6]-Dehydrogingerol (3b)**: yellow needles (59%), mp 123–124 °C (lit. 134–136 °C) [22]; UV (MeOH) λ_max_ (log ɛ) 341 (4.34), 270 (sh) (3.59), 247 (3.98) nm; IR (KBr) ν_max_ 3460, 3161, 2962, 2858, 1675, 1589, 1517, 1433, 1281, 1223, 1174, 1076, 872 cm^−1; 1^H-NMR (CDCl_3_) δ 7.50 (1H, d, *J =* 16.0 Hz, H-1), 7.11 (1H, dd, *J =* 8.0, 2.0 Hz, H-6′), 7.05 (1H, d, *J =* 2.0 Hz, H-2′), 6.93 (1H, d, *J =* 8.2 Hz, H-5′), 6.58 (1H, d, *J =* 16.0 Hz, H-2), 4.14 (1H, m, H-5), 3.92 (3H, s, -OCH_3_), 2.88 (1H, dd, *J =* 17.2, 3.2 Hz, H-4), 2.72 (1H, dd, *J =* 17.2, 8.6 Hz, H-4), 1.51–1.25 (8H, m, H-6~9), 0.89 (3H, t, *J =* 6.4 Hz, H-10); ^13^C-NMR (CDCl_3_) δ 200.1, 150.2, 148.7, 143.8, 127.6, 125.1, 124.1, 116.1, 111.4, 68.5, 56.2, 48.4, 38.0, 32.6, 26.0, 23.3, 14.3; EIMS *m*/*z* (*rel. int*.) 292 (M^+^, 51), 192 (20), 177 (100), 150 (47), 137 (40), 89 (10).

**[7]-Dehydroshogaol (2c)**: yellow syrup (13%); UV (MeOH) λ_max_ (log ɛ) 357 (3.91), 261 (3.95) nm; IR (neat) ν_max_ 3384, 2954, 2856, 1654, 1625, 1583, 1514, 1274, 1124, 1033 cm^−1; 1^H-NMR (CDCl_3_) δ 7.58 (1H, d, *J =* 15.8 Hz, H-1), 7.14 (1H, dd, *J =* 8.1, 1.8 Hz, H-6′), 7.08 (1H, d, *J =* 1.8 Hz, H-2′), 7.00 (1H, dt, *J =* 15.6, 6.9 Hz, H-5), 6.93 (1H, d, *J =* 8.1 Hz, H-5′), 6.81 (1H, d, *J =* 15.8 Hz, H-2), 6.43 (1H, dt, *J =* 15.6, 1.4 Hz, H-4), 5.92 (1H, br s, -OH), 3.94 (3H, s, -OCH_3_), 2.27 (2H, tdd, *J =* 6.9, 6.9, 1.4 Hz, H-6), 1.54–1.25 (8H, m, H-7~10), 0.89 (3H, t, *J =* 6.7 Hz, H-11); ^13^C-NMR (CDCl_3_) δ 189.3, 148.2, 148.0, 146.8, 143.4, 129.0, 127.3, 123.3, 122.7, 114.8, 109.7, 55.9, 32.7, 31.6, 28.9, 28.1, 22.5, 14.0; EIMS *m*/*z* (*rel. int*.) 288 (M^+^, 100), 287 (48), 217 (82), 204 (27), 177 (49), 137 (33); HREIMS *m*/*z* 288.1725 [M]^+^ (Calcd for C_18_H_24_O_3_, 288.1725).

**[7]-Dehydrogingerol (3c)**: yellow needles (56%), mp 108–109 °C (lit. 110–112 °C) [22]; UV (MeOH) λ_max_ (log ɛ) 340 (4.63), 271 (sh) (4.23), 252 (4.39) nm; IR (KBr) ν_max_ 3447, 3258, 2926, 2855, 1694, 1589, 1512, 1437, 1279, 1221, 1053, 812 cm^−1; 1^H-NMR (CDCl_3_) δ 7.46 (1H, d, *J =* 16.0 Hz, H-1), 7.04 (1H, dd, *J =* 8.2, 1.8 Hz, H-6′), 7.00 (1H, d, *J =* 1.8 Hz, H-2′), 6.88 (1H, d, *J =* 8.0 Hz, H-5′), 6.53 (1H, d, *J =* 16.0 Hz, H-2), 4.17–4.06 (1H, m, H-5), 3.87 (3H, s, -OCH_3_), 2.85 (1H, dd, *J =* 17.0, 3.2 Hz, H-4), 2.71 (1H, dd, *J =* 17.0, 8.6 Hz, H-4), 1.58–1.25 (10H, m, H-6~10), 0.85 (3H, t, *J =* 6.6 Hz, H-11); ^13^C-NMR (CDCl_3_) δ 201.0, 148.6, 147.0, 143.9, 126.7, 124.1, 123.7, 115.0, 109.5, 68.0, 56.0, 46.5, 36.6, 31.8, 29.3, 25.5, 22.6, 14.0; EIMS *m*/*z* (*rel. int*.) 306 (M^+^, 26), 217 (23), 192 (44), 177 (100), 150 (34), 145 (38), 137 (17), 89 (14).

**[8]-Dehydroshogaol (2d)**: yellow syrup (15%); UV (MeOH) λ_max_ (log ɛ) 357 (4.10), 258 (3.96) nm; IR(neat) ν_max_ 3395, 2925, 2856, 1660, 1614, 1581, 1514, 1278, 1174, 1033 cm^−1; 1^H-NMR (CDCl_3_) δ 7.58 (1H, d, *J =* 16.0 Hz, H-1), 7.12 (1H, dd, *J =* 8.2, 2.0 Hz, H-6′), 7.06 (1H, d, *J =* 2.0 Hz, H-2′), 6.99 (1H, dt, *J =* 15.6, 6.9 Hz, H-5), 6.93 (1H, d, *J =* 8.2 Hz, H-5′), 6.81 (1H, d, *J =* 16.0 Hz, H-2), 6.43 (1H, dt, *J =* 15.6, 1.4 Hz, H-4), 6.21 (1H, br s, -OH), 3.91 (3H, s, -OCH_3_), 2.26 (2H, tdd, *J =* 6.9, 6.9, 1.4 Hz, H-6), 1.52–1.27 (10H, m, H-7-11), 0.88 (3H, t, *J =* 6.8 Hz, H-12); ^13^C-NMR (CDCl_3_) δ 189.3, 148.2, 148.0, 146.8, 143.3, 129.0, 127.3, 123.2, 122.7, 114.8, 109.7, 55.9, 32.7, 31.7, 29.1, 29.0, 28.1, 22.6, 14.0; EIMS *m*/*z* (*rel. int*.) 302 (M^+^, 81), 301 (29), 217 (68), 177 (100), 150 (21), 137 (33), 55 (24); HREIMS *m*/*z* 302.1879 [M]^+^ (Calcd for C_19_H_26_O_3_, 302.1881).

**[8]-Dehydrogingerol (3d)**: yellow needles (66%), mp 83–84 °C (lit. 88–90 °C) [22]; UV (MeOH) λ_max_ (log ɛ) 340 (4.57), 270 (sh) (4.25), 247 (4.38) nm; IR (KBr) ν_max_ 3451, 3215, 2924, 2855, 1680, 1585, 1510, 1433, 1280, 1116, 854 cm^−1; 1^H-NMR (CDCl_3_) δ 7.51 (1H, d, *J =* 16.0 Hz, H-1), 7.10 (1H, dd, *J =* 8.2, 1.8 Hz, H-6′), 7.06 (1H, d, *J =* 1.8 Hz, H-2′), 6.93 (1H, d, *J =* 8.2 Hz, H-5′), 6.59 (1H, d, *J =* 16.0 Hz, H-2), 4.17–4.06 (1H, m, H-5), 3.94 (3H, s, -OCH_3_), 2.88 (1H, dd, *J =* 17.2, 3.1 Hz, H-4), 2.72 (1H, dd, *J =* 17.2, 8.7 Hz, H-4), 1.56–1.26 (12H, m, H-6~11), 0.88 (3H, t, *J =* 6.4 Hz, H-12); ^13^C-NMR (CDCl_3_) δ 200.2, 150.2, 148.8, 143.9, 127.6, 125.0, 124.2, 116.1, 111.5, 68.6, 56.2, 48.4, 38.1, 32.5, 30.3, 30.0, 26.3, 23.2, 14.3; EIMS *m*/*z* (*rel. int*.) 320 (M^+^, 22), 192 (53), 177 (100), 150 (28), 145 (31), 137 (30), 84 (37), 69 (28), 57 (40), 55(55); Anal. Calcd. for C_19_H_28_O_4_: C, 71.25%; H, 8.75%; Found: C, 71.26%; H, 8.79%.

**[9]-Dehydroshogaol (2e)**: yellow syrup (13%); UV (MeOH) λ_max_ (log ɛ) 355 (4.02), 260 (4.03) nm; IR (neat) ν_max_ 3358, 2925, 2856, 1641, 1587, 1525, 1274, 1120, 1037 cm^−1; 1^H-NMR (CDCl_3_) δ 7.57 (1H, d, *J =* 15.8 Hz, H-1), 7.11 (1H, dd, *J =* 8.2, 1.8 Hz, H-6′), 7.06 (1H, d, *J =* 1.8 Hz, H-2′), 6.99 (1H, dt, *J =* 15.4, 7.2 Hz, H-5), 6.91 (1H, d, *J =* 8.2 Hz, H-5′), 6.80 (1H, d, *J =* 15.8 Hz, H-2), 6.42 (1H, dt, *J =* 15.4, 1.3 Hz, H-4), 3.90 (3H, s, -OCH_3_), 2.25 (2H, tdd, *J =* 7.2, 6.8, 1.3 Hz, H-6), 1.41–1.26 (12H, m, H-7~12), 0.86 (3H, t, *J =* 6.6 Hz, H-13); ^13^C-NMR (CDCl_3_) δ 189.4, 148.3, 148.1, 146.9, 143.4, 129.0, 127.3, 123.3, 122.7, 114.9, 109.8, 56.0, 32.7, 31.8, 29.3, 29.2, 29.1, 28.2, 22.6, 14.1; EIMS *m*/*z* (*rel. int*.) 316 (M^+^, 100), 315 (36), 217 (83), 204 (23), 177 (86), 137 (44), 55 (21); HREIMS *m*/*z* 316.2040 [M]^+^ (Calcd for C_20_H_28_O_3_, 316.2038).

**[9]-Dehydrogingerol (3e)**: yellow needles (58%), mp 93–94 °C (lit. 93–94 °C) [22]; UV (MeOH) λ_max_ (log ɛ) 339 (4.50), 270 (sh) (4.10), 250 (4.27) nm; IR (KBr) ν_max_ 3451, 2926, 2854, 1676, 1583, 1516, 1460, 1280, 1170, 1031, 977, 810 cm^−1; 1^H-NMR (CDCl_3_) δ 7.50 (1H, d, *J =* 16.0 Hz, H-1), 7.10 (1H, dd, *J =* 8.2, 1.8 Hz, H-6′), 7.05 (1H, d, *J =* 1.8 Hz, H-2′), 6.93 (1H, d, *J =* 8.2 Hz, H-5′), 6.59 (1H, d, *J =* 16.0 Hz, H-2), 4.17–4.06 (1H, m, H-5), 3.94 (3H, s, -OCH_3_), 2.87 (1H, dd, *J =* 17.1, 3.0 Hz, H-4), 2.72 (1H, dd, *J =* 17.1, 8.7 Hz, H-4), 1.56–1.28 (14H, m, H-6~12), 0.88 (3H, t, *J =* 6.4 Hz, H-13); ^13^C-NMR (CDCl_3_) δ 200.1, 150.1, 148.7, 143.8, 127.6, 125.0, 124.1, 116.1, 111.4, 68.5, 56.2, 48.4, 38.0, 32.6, 30.4, 30.3, 30.0, 26.3, 23.2, 14.3; EIMS *m*/*z* (*rel. int*.) 334 (M^+^, 33), 316 (27), 217 (22), 192 (50), 177 (100), 150 (30), 145 (27), 137 (46), 57 (20).

**[10]-Dehydroshogaol (2f)**: yellow syrup (6%); UV (MeOH) λ_max_ (log ɛ) 355 (4.18), 257 (4.04) nm; IR (neat) ν_max_ 3533, 2925, 2856, 1660, 1614, 1581, 1514, 1278, 1201, 1120, 1031 cm^−1; 1^H-NMR (CDCl_3_) δ 7.58 (1H, d, *J =* 15.9 Hz, H-1), 7.13 (1H, dd, *J =* 8.2, 1.8 Hz, H-6′), 7.06 (1H, d, *J =* 1.8 Hz, H-2′), 6.99 (1H, dt, *J =* 15.6, 6.8 Hz, H-5), 6.91 (1H, d, *J =* 8.2 Hz, H-5′), 6.81 (1H, d, *J =* 15.9 Hz, H-2), 6.42 (1H, dt, *J =* 15.6, 1.4 Hz, H-4), 6.17 (1H, br s, -OH), 3.92 (3H, s, -OCH_3_), 2.25 (2H, tdd, *J =* 6.8, 6.8, 1.4 Hz, H-6), 1.52–1.26 (14H, m, H-7~13), 0.87 (3H, t, *J =* 6.7 Hz, H-14); ^13^C-NMR (CDCl_3_) δ 189.3, 148.2, 148.0, 146.8, 143.4, 129.0, 127.3, 123.3, 122.7, 114.8, 109.7, 55.9, 32.7, 31.8, 29.5, 29.4, 29.3, 29.2, 28.2, 22.7, 14.1; EIMS *m*/*z* (*rel. int*.) 330 (M^+^, 47), 217 (37), 177 (100), 152 (53), 150 (22), 137 (35), 97 (26), 85 (23), 71 (36), 57 (80), 55 (61); HREIMS *m*/*z* 330.2196 [M]^+^ (Calcd for C_21_H_30_O_3_, 330.2194).

**[10]-Dehydrogingerol (3f)**: yellow needles (50%), mp 74–75 °C (lit. 76–77.5 °C) [22]; UV (MeOH) λ_max_ (log ɛ) 340 (4.27), 273 (sh) (3.68), 239 (4.06) nm; IR (KBr) ν_max_ 3414, 2926, 2855, 1656, 1587, 1515, 1460, 1281, 1169, 1031, 979, 810 cm^−1; 1^H-NMR (CDCl_3_) δ 7.51 (1H, d, *J =* 16.1 Hz, H-1), 7.11 (1H, dd, *J =* 8.1, 1.8 Hz, H-6′), 7.05 (1H, d, *J =* 1.8 Hz, H-2′), 6.93 (1H, d, *J =* 8.1 Hz, H-5′), 6.59 (1H, d, *J =* 16.1 Hz, H-2), 5.97 (1H, br s, -OH), 4.17–4.06 (1H, m, H-5), 3.93 (3H, s, -OCH_3_), 2.88 (1H, dd, *J =* 17.1, 3.0 Hz, H-4), 2.73 (1H, dd, *J =* 17.1, 8.7 Hz, H-4), 1.57–1.27 (16H, m, H-6~13), 0.88 (3H, t, *J =* 6.7 Hz, H-14); ^13^C-NMR (CDCl_3_) δ 200.9, 148.4, 146.8, 143.8, 126.7, 124.1, 123.7, 114.8, 109.4, 68.0, 55.9, 46.5, 36.5, 31.8, 29.5 (×2), 29.4, 29.3, 25.5, 22.6, 14.1; EIMS *m*/*z* (*rel. int*.) 348 (M^+^, 24), 232 (21), 192 (17), 177 (52), 150 (76), 145 (12), 137 (29), 97 (29), 91 (45), 57 (100).

#### 3.2.3. General Procedure for the Synthesis of [*n*]-Epoxydehydroparadol (**7a**–**f**)

A 1.0 M THF solution of lithium bis(trimethylsilyl)amide (20.8 mL) was added dropwise to a solution of dehydrozingerone (**9**) (2.0 g, 10.4 mmol) in dry THF (10 mL) at 0 °C in an air atmosphere. After the mixture had been stirred for 1 h, the appropriate aldehyde (31.4 mmol) was added and stirred for 3 h. The reaction was then quenched with 5% HCl_(aq)_ at 0 °C and extracted with EtOAc (4 × 20 mL). The organic layers were combined, washed with brine, dried over Na_2_SO_4_, and concentrated under reduced pressure. Products **2** and **7** were isolated using C-18 gel column chromatography (water/methanol = 1/2).

**1-(4-Hydroxy-3-methoxyphenyl)-4,5-expoxynon-1-en-3-one (7a)**: yellow syrup (8%); UV (MeOH) λ_max_ (log ɛ) 349 (4.09), 251 (3.75) nm; IR (KBr) ν_max_ 3451, 2956, 2931, 1693, 1587, 1514, 1465, 1271, 1031 cm^−1; 1^H-NMR (CDCl_3_) δ 7.71 (1H, d, *J =* 16.0 Hz, H-1), 7.13 (1H, dd, *J =* 8.2, 1.8 Hz, H-6′), 7.06 (1H, d, *J =* 1.8 Hz, H-2′), 6.92 (1H, d, *J =* 8.2 Hz, H-5′), 6.71 (1H, d, *J =* 16.0 Hz, H-2), 6.01 (1H, br s, -OH), 3.92 (3H, s, -OCH_3_), 3.41 (1H, d, *J =* 2.0 Hz, H-4), 3.11 (1H, td, *J =* 5.3, 2.0 Hz, H-5), 1.74–1.25 (6H, m, H-6~8), 0.92 (3H, t, *J =* 6.8 Hz, H-9); ^13^C-NMR (CDCl_3_) δ 195.7, 148.7, 146.8, 145.2, 126.8, 124.2, 116.8, 114.8, 109.7, 59.6, 58.4, 56.0, 31.6, 27.9, 22.4, 13.9; EIMS *m*/*z* (*rel. int*.) 276 (M^+^, 38), 177 (100), 145 (20); HREIMS *m*/*z* 276.1363 [M]^+^ (Calcd for C_16_H_20_O_4_, 276.1361).

**1-(4-Hydroxy-3-methoxyphenyl)-4,5-expoxydec-1-en-3-one (7b)**: yellow syrup (10%); UV (MeOH) λ_max_ (log ɛ) 354 (4.27), 251 (3.93) nm; IR (neat) ν_max_ 3414, 2954, 2862, 1676, 1585, 1512, 1460, 1272, 1031 cm^−1; 1^H-NMR (CDCl_3_) δ 7.71 (1H, d, *J =* 15.9 Hz, H-1), 7.13 (1H, dd, *J =* 8.1, 1.8 Hz, H-6′), 7.06 (1H, d, *J =* 1.8 Hz, H-2′), 6.91 (1H, d, *J =* 8.1 Hz, H-5′), 6.71 (1H, d, *J =* 15.9 Hz, H-2), 3.92 (3H, s, -OCH_3_), 3.41 (1H, d, *J =* 2.0 Hz, H-4), 3.12 (1H, td, *J =* 5.2, 2.0 Hz, H-5), 1.73–1.24 (8H, m, H-6~9), 0.89 (3H, t, *J =* 6.8 Hz, H-10); ^13^C-NMR (CDCl_3_) δ 195.6, 148.6, 146.7, 145.2, 126.8, 124.2, 116.8, 114.8, 109.7, 59.5, 58.4, 56.0, 31.8, 31.4, 25.4, 22.4, 13.9; EIMS *m*/*z* (*rel. int*.) 290 (M^+^, 39), 178 (21), 177 (100), 145 (22); HREIMS *m*/*z* 290.1516 [M]^+^ (Calcd for C_17_H_22_O_4_, 290.1518).

**1-(4-Hydroxy-3-methoxyphenyl)-4,5-expoxyundec-1-en-3-one (7c)**: yellow syrup (9%); UV (MeOH) λ_max_ (log ɛ) 352 (4.19), 254 (3.89) nm; IR (neat) ν_max_ 3408, 2927, 2858, 1672, 1585, 1514, 1434, 1276, 1031 cm^−1; 1^H-NMR (CDCl_3_) δ 7.71 (1H, d, *J =* 15.8 Hz, H-1), 7.13 (1H, dd, *J =* 8.4, 2.0 Hz, H-6′), 7.06 (1H, d, *J =* 2.0 Hz, H-2′), 6.91 (1H, d, *J =* 8.4 Hz, H-5′), 6.70 (1H, d, *J =* 15.8 Hz, H-2), 6.05 (1H, br s, -OH), 3.92 (3H, s, -OCH_3_), 3.41 (1H, d, *J =* 2.0 Hz, H-4), 3.13 (1H, td, *J =* 5.0, 2.0 Hz, H-5), 1.73–1.29 (10H, m, H-6~10), 0.88 (3H, t, *J =* 6.8 Hz, H-11); ^13^C-NMR (CDCl_3_) δ 195.6, 148.6, 146.7, 145.2, 126.8, 124.2, 116.8, 114.8, 109.7, 59.5, 58.4, 56.0, 31.8, 31.6, 28.9, 25.7, 22.4, 13.9 ; EIMS *m*/*z* (*rel. int*.) 304 (M^+^, 46), 178 (26), 177 (100), 145 (26); HREIMS *m*/*z* 304.1673 [M]^+^ (Calcd for C_18_H_24_O_4_, 304.1674).

**1-(4-Hydroxy-3-methoxyphenyl)-4,5-expoxydodec-1-en-3-one (7d)**: yellow syrup (9%); UV (MeOH) λ_max_ (log ɛ) 351 (4.33), 255 (4.07) nm; IR(neat) ν_max_ 3395, 2925, 2858, 1672, 1581, 1514, 1434, 1172, 1031 cm^−1; 1^H-NMR (CDCl_3_) δ 7.70 (1H, d, *J =* 16.0 Hz, H-1), 7.12 (1H, dd, *J =* 8.2, 2.0 Hz, H-6′), 7.05 (1H, d, *J =* 2.0 Hz, H-2′), 6.90 (1H, d, *J =* 8.2 Hz, H-5′), 6.70 (1H, d, *J =* 16.0 Hz, H-2), 6.16 (1H, br s, -OH), 3.91 (3H, s, -OCH_3_), 3.41 (1H, d, *J =* 2.0 Hz, H-4), 3.12 (1H, td, *J =* 5.2, 2.0 Hz, H-5), 1.73–1.26 (12H, m, H-6~11), 0.87 (3H, t, *J =* 6.8 Hz, H-12); ^13^C-NMR (CDCl_3_) δ 195.7, 148.7, 146.8, 145.2, 126.8, 124.2, 116.8, 114.8, 109.7, 59.5, 58.4, 56.0, 31.8, 31.7, 29.2, 29.1, 25.8, 22.6, 14.0; EIMS *m*/*z* (*rel. int*.) 318 (M^+^, 37), 178 (22), 177 (100), 145 (19); HREIMS *m*/*z* 318.1834 [M]^+^ (Calcd for C_19_H_26_O_4_, 318.1831).

**1-(4-Hydroxy-3-methoxyphenyl)-4,5-expoxytridec-1-en-3-one (7e)**: yellow syrup (8%); UV (MeOH) λ_max_ (log ɛ) 353 (4.32), 254 (4.04) nm; IR (neat) ν_max_ 3404, 2925, 2856, 1672, 1583, 1514, 1434, 1276, 1031 cm^−1; 1^H-NMR (CDCl_3_) δ 7.72 (1H, d, *J =* 15.8 Hz, H-1), 7.14 (1H, dd, *J =* 8.2, 1.8 Hz, H-6′), 7.07 (1H, d, *J =* 1.8 Hz, H-2′), 6.92 (1H, d, *J =* 8.2 Hz, H-5′), 6.71 (1H, d, *J =* 15.8 Hz, H-2), 5.95 (1H, br s, -OH), 3.94 (3H, s, -OCH_3_), 3.41 (1H, d, *J =* 2.0 Hz, H-4), 3.13 (1H, td, *J =* 5.0, 2.0 Hz, H-5), 1.74–1.27 (14H, m, H-6~12), 0.88 (3H, t, *J =* 6.8 Hz, H-13); ^13^C-NMR (CDCl_3_) δ 195.6, 148.7, 146.8, 145.1, 126.9, 124.2, 116.9, 114.8, 109.7, 59.6, 58.4, 56.0, 31.8 (×2), 29.4, 29.3, 29.1, 25.8, 22.6, 14.0; EIMS *m*/*z* (*rel. int*.) 332 (M^+^, 36), 177 (100), 145 (15), 55 (12); HREIMS *m*/*z* 332.1990 [M]^+^ (Calcd for C_20_H_28_O_4_, 332.1987).

**1-(4-Hydroxy-3-methoxyphenyl)-4,5-expoxytetradec-1-en-3-one (7f)**: yellow syrup (8%); UV (MeOH) λ_max_ (log ɛ) 350 (4.19), 253 (4.17) nm; IR (neat) ν_max_ 3423, 2925, 2854, 1676, 1585, 1512, 1460, 1274, 1031 cm^−1; 1^H-NMR (CDCl_3_) δ 7.71 (1H, d, *J =* 15.8 Hz, H-1), 7.14 (1H, dd, *J =* 8.2, 1.8 Hz, H-6′), 7.07 (1H, d, *J =* 1.8 Hz, H-2′), 6.92 (1H, d, *J =* 8.2 Hz, H-5′), 6.72 (1H, d, *J =* 15.8 Hz, H-2), 5.90 (1H, br s, -OH), 3.94 (3H, s, -OCH_3_), 3.41 (1H, d, *J =* 2.0 Hz, H-4), 3.13 (1H, td, *J =* 5.4, 2.0 Hz, H-5), 1.74–1.27 (16H, m, H-6~13), 0.88 (3H, t, *J =* 6.8 Hz, H-14); ^13^C-NMR (CDCl_3_) δ 195.7, 148.7, 146.8, 145.2, 126.9, 124.2, 116.8, 114.8, 109.7, 59.6, 58.4, 56.0, 31.9, 31.8, 29.5, 29.4, 29.3, 29.2, 25.8, 22.6, 14.1; EIMS *m*/*z* (*rel. int*.) 346 (M^+^, 33), 177 (100), 151 (23), 150 (55), 55 (20); HREIMS *m*/*z* 346.2145 [M]^+^ (Calcd for C_21_H_30_O_4_, 346.2144).

#### 3.2.4. General Procedure for the Synthesis of [*n*]-Paradols (**5a**–**f**)

A solution of [*n*]-dehydroshogaols (**2a**–**f**) (0.96 mmol) in ethyl acetate (20 mL) containing palladium-charcoal (5%, 0.05 g) was stirred under hydrogen at atmospheric pressure and room temperature for 30 min. The reaction mixture was monitored by TLC until no starting material remained. The catalyst was removed through celite, and the filtrate was concentrated under reduced pressure. The product was isolated using silica gel column chromatography (EtOAc/hexanes = 1/4).

**[5]-Paradol (5a)**: colorless syrup (79%) [23]; UV (MeOH) λ_max_ (log ɛ) 282 (3.41) nm; IR (neat) ν_max_ 3439, 2939, 2862, 1707, 1608, 1516, 1452, 1365, 1269, 1031, 806 cm^−1; 1^H-NMR (CDCl_3_) δ 6.79 (1H, d, *J =* 8.0 Hz, H-5′), 6.67 (1H, d, *J =* 1.8 Hz, H-2′), 6.63 (1H, dd, *J =* 8.0, 1.8 Hz, H-6′), 3.82 (3H, s, -OCH_3_), 2.84–2.63 (4H, m, H-1, -2), 2.35 (2H, t, *J =* 7.2 Hz, H-4), 1.58–1.51 (2H, m, H-5), 1.23 (6H, m, H-6~8), 0.87 (3H, t, *J =* 6.2 Hz, H-9); ^13^C-NMR (CDCl_3_) δ 210.8, 146.4, 143.8, 132.9, 120.6, 114.3, 111.1, 55.7, 44.5, 43.0, 31.5, 29.4, 28.8, 23.7, 22.4, 14.0; EIMS *m*/*z* (*rel. int*.) 264 (M^+^, 58), 179 (19), 151 (22), 137 (100); HREIMS *m*/*z* 246.1729 [M]^+^ (Calcd for C_16_H_24_O_3_, 246.1725).

**[6]-Paradol (5b)**: colorless syrup (78%) [23]; UV (MeOH) λ_max_ (log ɛ) 281 (3.34) nm; IR (neat) ν_max_ 3451, 2940, 2862, 1713, 1516, 1452, 1367, 1267, 1036, 804 cm^−1; 1^H-NMR (CDCl_3_) δ 6.80 (1H, d, *J =* 8.0 Hz, H-5′), 6.67 (1H, d, *J =* 1.8 Hz, H-2′), 6.64 (1H, dd, *J =* 8.0, 1.8 Hz, H-6′), 3.85 (3H, s, -OCH_3_), 2.86–2.63 (4H, m, H-1, -2), 2.36 (2H, t, *J =* 7.4 Hz, H-4), 1.58–1.51 (2H, m, H-5), 1.24 (8H, m, H-6~9), 0.88 (3H, t, *J =* 6.2 Hz, H-10); ^13^C-NMR (CDCl_3_) δ 210.6, 146.3, 143.8, 133.1, 120.7, 114.3, 111.0, 55.8, 44.6, 43.1, 31.6, 29.5, 29.1, 29.0, 23.8, 22.5, 14.0; EIMS *m*/*z* (*rel. int*.) 278 (M^+^, 67), 179 (21), 151 (23), 137 (100), 117 (19), 99 (23), 55 (21); HREIMS *m*/*z* 278.1883 [M]^+^ (Calcd for C_17_H_26_O_3_, 278.1881).

**[7]-Paradol (5c)**: colorless syrup (81%) [23]; UV (MeOH) λ_max_ (log ɛ) 282 (3.45) nm; IR (neat) ν_max_ 3543, 2930, 2858, 1707, 1608, 1516, 1452, 1365, 1269, 1034, 806 cm^−1; 1^H-NMR (CDCl_3_) δ 6.77 (1H, d, *J =* 8.0 Hz, H-5′), 6.66 (1H, d, *J =* 1.8 Hz, H-2′), 6.61 (1H, dd, *J =* 8.0, 1.8 Hz, H-6′), 5.48 (1H, br s, -OH), 3.79 (3H, s, -OCH_3_), 2.83–2.61 (4H, m, H-1, -2), 2.33 (2H, t, *J =* 7.2 Hz, H-4), 1.58–1.51 (2H, m, H-5), 1.22 (10H, m, H-6~10), 0.85 (3H, t, *J =* 6.8 Hz, H-11); ^13^C-NMR (CDCl_3_) δ 210.7, 146.5, 143.9, 132.9, 120.6, 114.4, 111.1, 55.7, 44.4, 42.9, 31.7, 29.4, 29.2, 29.1, 29.0, 23.7, 22.5, 14.0; EIMS *m*/*z* (*rel. int*.) 292 (M^+^, 36), 179 (17), 151 (21), 137 (100), 119 (10), 55 (11).

**[8]-Paradol (5d)**: colorless powder (77%), mp 42–43 °C (lit. 42–43 °C) [23]; UV (MeOH) λ_max_ (log ɛ) 282 (3.46) nm; IR (KBr) ν_max_ 3541, 2920, 2856, 1707, 1608, 1514, 1365, 1271, 1030, 806 cm^−1; 1^H-NMR (CDCl_3_) δ 6.82 (1H, d, *J =* 7.8 Hz, H-5′), 6.68–6.63 (2H, m, H-2′, -6′), 3.86 (3H, s, -OCH_3_), 2.87–2.64 (4H, m, H-1, -2), 2.37 (2H, t, *J =* 7.2 Hz, H-4), 1.58–1.51 (2H, m, H-5), 1.25 (12H, m, H-6~11), 0.88 (3H, t, *J =* 6.8 Hz, H-12); ^13^C-NMR (CDCl_3_) δ 210.6, 146.4, 143.8, 133.1, 120.7, 114.3, 111.0, 55.8, 44.6, 43.1, 31.8, 29.5, 29.3 (×3), 29.2, 23.8, 22.6, 14.1; EIMS *m*/*z* (*rel. int*.) 306 (M^+^, 17), 292 (12), 164 (21), 179 (19), 151 (22), 137 (100), 57 (10); Anal. Calcd. for C_19_H_30_O_3_: C, 74.50%; H, 9.80%; Found: C, 74.57%; H, 9.84%.

**[9]-Paradol (5e)**: colorless powder (80%), mp 49–50 °C (lit. 48–49 °C) [23]; UV (MeOH) λ_max_ (log ɛ) 282 (3.41) nm; IR (KBr) ν_max_ 3516, 2922, 2856, 1712, 1608, 1516, 1361, 1273, 1165, 1028, 856 cm^−1; 1^H-NMR (CDCl_3_) δ 6.81 (1H, d, *J =* 8.0 Hz, H-5′), 6.68–6.63 (2H, m, H-2′, -6′), 3.86 (3H, s, -OCH_3_), 2.86–2.64 (4H, m, H-1, -2), 2.36 (2H, t, *J =* 7.2 Hz, H-4), 1.58–1.51 (2H, m, H-5), 1.24 (14H, m, H-6~12), 0.87 (3H, t, *J =* 6.8 Hz, H-13); ^13^C-NMR (CDCl_3_) δ 210.6, 146.3, 143.8, 133.1, 120.7, 114.2, 111.0, 56.0, 44.5, 43.1, 31.8, 29.5 (×2), 29.4 29.3, 29.2, 29.1, 23.8, 22.6, 14.1; EIMS *m*/*z* (*rel. int*.) 320 (M^+^, 80), 179 (19), 151 (21), 137 (100), 119 (8); Anal. Calcd. for C_20_H_32_O_3_: C, 75.00%; H, 10.00%; Found: C, 75.01%; H, 10.01%.

**[10]-Paradol (5f)**: colorless powder (79%), mp 50–51 °C (lit. 50–51 °C) [23]; UV (MeOH) λ_max_ (log ɛ) 280 (3.44) nm; IR (KBr) ν_max_ 3486, 2920, 2856, 1707, 1608, 1512, 1361, 1273, 1165, 1028, 856 cm^−1; 1^H-NMR (CDCl_3_) δ 6.81 (1H, d, *J =* 8.0 Hz, H-5′), 6.68–6.63 (2H, m, H-2′, -6′), 3.85 (3H, s, -OCH_3_), 2.86–2.64 (4H, m, H-1, -2), 2.37 (2H, t, *J =* 7.2 Hz, H-4), 1.58–1.51 (2H, m, H-5), 1.25 (16H, m, H-6~13), 0.88 (3H, t, *J =* 6.6 Hz, H-14); ^13^C-NMR (CDCl_3_) δ 210.6, 146.4, 143.8, 133.0, 120.6, 114.3, 111.0, 55.7, 44.5, 43.0, 31.8, 29.5 (×2), 29.4 29.3 (×2), 29.2, 29.1, 23.7, 22.6, 14.0; Anal. Calcd. for C_21_H_34_O_3_: C, 75.45%; H, 10.18%; Found: C, 75.49%; H, 10.13%.

#### 3.2.5. General Procedure for the Synthesis of [*n*]-Dehydroparadols (**6a**–**f**)

A solution of [*n*]-dehydroshogaols (**2a**–**f**) (0.96 mmol) in ethyl acetate (20 mL) containing palladium-charcoal (5%, 0.015 g) was stirred under hydrogen at atmospheric pressure and room temperature for 40 min. The reaction mixture was monitored using thin layer chromatography (TLC) until no starting material remained. The catalyst was removed through celite, and the filtrate was concentrated under reduced pressure conditions. The product was isolated using silica gel column chromatography (EtOAc/hexanes = 1/3).

**[5]-Dehydroparadol (6a)**: colorless powder (80%), mp 52–53 °C (lit. 52–53 °C) [23]; UV (MeOH) λ_max_ (log ɛ) 340 (4.16), 224 (3.79) nm; IR (KBr) ν_max_ 3400, 2930, 2860, 1666, 1587, 1514, 1460, 1375, 1276, 1033, 979, 812 cm^−1; 1^H-NMR (CDCl_3_) δ 7.48 (1H, d, *J =* 16.1 Hz, H-1), 7.09 (1H, dd, *J =* 8.1, 2.0 Hz, H-6′), 7.05 (1H, d, *J =* 2.0 Hz, H-2′), 6.92 (1H, d, *J =* 8.1 Hz, H-5′), 6.59 (1H, d, *J =* 16.1 Hz, H-2), 6.12 (1H, br s, -OH), 3.92 (3H, s, -OCH_3_), 2.64 (2H, t, *J =* 7.1 Hz, H-4), 1.70–1.59 (2H, m, H-5), 1.41–1.22 (6H, m, H-6~8), 0.88 (3H, t, *J =* 6.5 Hz, H-9); ^13^C-NMR (CDCl_3_) δ 200.8, 148.1, 146.8, 142.6, 127.1, 124.1, 123.3, 114.8, 109.4, 55.9, 40.7, 31.6, 29.0, 24.5, 22.5, 14.0; EIMS *m/z* (*rel. int*.) 262 (M^+^, 31), 192 (34), 177 (100), 145 (22), 137 (44), 117 (10), 89 (12).

**[6]-Dehydroparadol (6b)**: colorless powder (76%), mp 47–48 °C (lit. 44–45 °C) [23]; UV (MeOH) λ_max_ (log ɛ) 341 (4.04), 224 (3.85) nm; IR (KBr) ν_max_ 3400, 2926, 2856, 1666, 1601, 1514, 1460, 1375, 1278, 1031, 979, 810 cm^−1; 1^H-NMR (CDCl_3_) δ 7.47 (1H, d, *J =* 16.0 Hz, H-1), 7.08 (1H, dd, *J =* 8.1, 1.8 Hz, H-6′), 7.04 (1H, d, *J =* 1.8 Hz, H-2′), 6.91 (1H, d, *J =* 8.1 Hz, H-5′), 6.58 (1H, d, *J =* 16.0 Hz, H-2), 6.19 (1H, br s, -OH), 3.90 (3H, s, -OCH_3_), 2.63 (2H, t, *J =* 7.2 Hz, H-4), 1.69–1.59 (2H, m, H-5), 1.31–1.27 (8H, m, H-6~9), 0.88 (3H, t, *J =* 6.6 Hz, H-10); ^13^C-NMR (CDCl_3_) δ 200.8, 148.1, 146.8, 142.6, 126.9, 123.9, 123.3, 114.8, 109.4, 55.9, 40.6, 31.6, 29.3, 29.0, 24.5, 22.5, 14.0; EIMS *m/z* (*rel. int*.) 276 (M^+^, 31), 192 (40), 177 (100), 145 (19), 137 (71), 117 (10), 89 (11), 55(10); HREIMS *m/z* 276.1727 [M]^+^ (Calcd for C_17_H_24_O_3_, 276.1725).

**[7]-Dehydroparadol (6c)**: colorless powder (75%), mp 49–50 °C (lit. 45–46 °C) [23]; UV (MeOH) λ_max_ (log ɛ) 338 (4.03), 225 (3.80) nm; IR (KBr) ν_max_ 3401, 2926, 2854, 1676, 1589, 1514, 1460, 1377, 1207, 1033, 979, 810 cm^−1; 1^H-NMR (CDCl_3_) δ 7.46 (1H, d, *J =* 16.1 Hz, H-1), 7.05 (1H, dd, *J =* 8.0, 1.8 Hz, H-6′), 7.01 (1H, d, *J =* 1.8 Hz, H-2′), 6.89 (1H, d, *J =* 8.0 Hz, H-5′), 6.57 (1H, d, *J =* 16.1 Hz, H-2), 3.86 (3H, s, -OCH_3_), 2.61 (2H, t, *J =* 7.2 Hz, H-4), 1.67–1.57 (2H, m, H-5), 1.26–1.23 (10H, m, H-6~10), 0.85 (3H, t, *J =* 6.8 Hz, H-11); ^13^C-NMR (CDCl_3_) δ 200.9, 148.3, 146.9, 142.8, 126.8, 123.8, 123.3, 114.9, 109.5, 55.8, 40.5, 31.7, 29.3 (×2), 29.1, 24.5, 22.6, 14.0; EIMS *m/z* (*rel. int*.) 290 (M^+^, 15), 205 (12), 192 (28), 177 (63), 137 (100), 91 (11), 55(12); HREIMS *m/z* 290.1885 [M]^+^ (Calcd for C_18_H_26_O_3_, 290.1881).

**[8]-Dehydroparadol (6d)**: colorless powder (73%), mp 58–59 °C (lit. 57–58 °C) [23]; UV (MeOH) λ_max_ (log ɛ) 339 (4.09), 223 (3.96) nm; IR (KBr) ν_max_ 3401, 2925, 2854, 1675, 1589, 1514, 1460, 1272, 1033, 979, 810 cm^−1; 1^H-NMR (CDCl_3_) δ 7.48 (1H, d, *J =* 16.0 Hz, H-1), 7.06 (1H, dd, *J =* 8.0, 1.8 Hz, H-6′), 7.04 (1H, d, *J =* 1.8 Hz, H-2′), 6.91 (1H, d, *J =* 8.0 Hz, H-5′), 6.59 (1H, d, *J =* 16.0 Hz, H-2), 6.19 (1H, br s, -OH), 3.91 (3H, s, -OCH_3_), 2.63 (2H, t, *J =* 7.0 Hz, H-4), 1.67–1.62 (2H, m, H-5), 1.28–1.25 (12H, m, H-6~11), 0.86 (3H, t, *J =* 6.8 Hz, H-12); ^13^C-NMR (CDCl_3_) δ 200.8, 148.1, 146.8, 142.7, 127.0, 123.9, 123.3, 114.8, 109.4, 55.9, 40.6, 31.8, 29.4 (×2), 29.3, 29.2, 24.5, 22.6, 14.0; EIMS *m/z* (*rel. int*.) 304 (M^+^, 27), 205 (13), 192 (34), 177 (66), 151 (18), 137 (100), 91 (10), 55(12); Anal. Calcd for C_19_H_28_O_3_: C, 75.00%; H, 9.21%; Found: C, 74.99%; H, 9.25%.

**[9]-Dehydroparadol (6e)**: colorless powder (74%), mp 56–58 °C (lit. 53–54 °C) [23]; UV (MeOH) λ_max_ (log ɛ) 337 (3.96), 224 (3.74) nm; IR (KBr) ν_max_ 3395, 2925, 2854, 1666, 1589, 1516, 1460, 1277, 1033, 979, 812 cm^−1; 1^H-NMR (CDCl_3_) δ 7.47 (1H, d, *J =* 16.0 Hz, H-1), 7.08 (1H, dd, *J =* 8.0, 1.8 Hz, H-6′), 7.03 (1H, d, *J =* 1.8 Hz, H-2′), 6.90 (1H, d, *J =* 8.0 Hz, H-5′), 6.58 (1H, d, *J =* 16.0 Hz, H-2), 6.22 (1H, br s, -OH), 3.90 (3H, s, -OCH_3_), 2.63 (2H, t, *J =* 7.2 Hz, H-4), 1.69–1.62 (2H, m, H-5), 1.28–1.24 (14H, m, H-6~12), 0.86 (3H, t, *J =* 6.6 Hz, H-13); ^13^C-NMR (CDCl_3_) δ 200.8, 148.1, 146.8, 142.6, 126.9, 123.9, 123.3, 114.8, 109.4, 55.8, 40.6, 31.8, 29.5, 29.4, 29.3 (×2), 29.2, 24.5, 22.5, 14.0; EIMS *m/z* (*rel. int*.) 318 (M^+^, 27), 192 (57), 177 (100), 153 (22), 137 (39), 55(23).

**[10]-Dehydroparadol (6f)**: colorless powder (79%), mp 72–73 °C (lit. 76–77 °C) [23]; UV (MeOH) λ_max_ (log ɛ) 339 (3.99), 225 (3.78) nm; IR(KBr) ν_max_ 3412, 2920, 2854, 1666, 1589, 1512, 1460, 1277, 1033 cm^−1; 1^H-NMR (CDCl_3_) δ 7.47 (1H, d, *J =* 16.0 Hz, H-1), 7.10–7.04 (2H, m, H-2′,6′), 6.89 (1H, d, *J =* 8.2 Hz, H-5′), 6.58 (1H, d, *J =* 16.0 Hz, H-2), 6.25 (1H, br s, -OH), 3.90 (3H, s, -OCH_3_), 2.63 (2H, t, *J =* 7.2 Hz, H-4), 1.69–1.59 (2H, m, H-5), 1.28–1.25 (16H, m, H-6~13), 0.86 (3H, t, *J =* 6.4 Hz, H-14); ^13^C-NMR (CDCl_3_) δ 200.8, 148.1, 146.8, 142.6, 126.9, 123.9, 123.3, 114.8, 109.4, 55.8, 40.5, 31.8, 29.5 (×2), 29.4, 29.3 (×2), 29.2, 24.5, 22.6, 14.0.

#### 3.2.6. General Procedure for the Synthesis of [*n*]-Gingerols (**8a**–**f**)

A solution of [*n*]-dehydrogingerols (**3a**–**f**) (1.1 mmol) in ethyl acetate (20 mL) containing palladium-charcoal (5%, 0.04 g) was stirred under hydrogen at atmospheric pressure and room temperature for 40 min. The reaction mixture was monitored using TLC until no starting material remained. The catalyst was removed through celite, and the filtrate was concentrated under reduced pressure. The product was isolated using silica gel column chromatography (EtOAc/hexanes = 1/2).

**[5]-Gingerol (8a)**: colorless powder (85%), mp 44–45 °C (lit. 45–46 °C); UV (MeOH) λ_max_ (log ɛ) 282 (3.44), 224 (3.85) nm; IR (KBr) ν_max_ 3460, 2943, 2864, 1704, 1612, 1138, 1371, 1271, 1138, 1034, 806 cm^−1; 1^H-NMR (CDCl_3_) δ 6.78 (1H, d, *J =* 7.8 Hz, H-5′), 6.64 (1H, d, *J =* 1.8 Hz, H-2′), 6.61 (1H, dd, *J =* 7.8, 1.8 Hz, H-6′), 4.34 (1H, br s, -OH), 4.06–3.94 (1H, m, H-5), 3.81 (3H, s, -OCH_3_), 2.48–2.65 (4H, m, H-1, -2), 2.52–2.48 (2H, m, H-4), 1.50–1.22 (6H, m, H-6~8), 0.86 (3H, t, *J =* 7.0 Hz, H-9); ^13^C-NMR (CDCl_3_) δ 211.4, 147.0, 144.0, 132.6, 120.7, 114.4, 111.0, 67.6, 55.9, 49.3, 45.4, 36.1, 29.2, 27.6, 22.6, 14.3; EIMS *m*/*z* (*rel. int*.) 280 (M^+^, 31), 205 (9), 150 (50), 137 (100), 91 (10); HREIMS *m*/*z* 280.1677 [M]^+^ (Calcd for C_16_H_24_O_4_, 280.1674).

**[6]-Gingerol (8b)**: colorless syrup (84%); UV (MeOH) λ_max_ (log ɛ) 282 (3.51) nm; IR (KBr) ν_max_ 3469, 2937, 2860, 1705, 1608, 1516, 1371, 1140, 1036, 806 cm^−1; 1^H-NMR (CDCl_3_) δ 6.79 (1H, d, *J =* 7.8 Hz, H-5′), 6.66–6.60 (2H,m, H-2′, -6′), 4.05–3.99 (1H, m, H-5), 3.83 (3H, s, -OCH_3_), 2.86–2.66 (4H, m, H-1, -2), 2.53–2.48 (2H, m, H-4), 1.49–1.24 (8H, m, H-6~9), 0.86 (3H, t, *J =* 6.2 Hz, H-10); ^13^C-NMR (CDCl_3_) δ 211.4, 146.4, 143.9, 132.5, 120.6, 114.4, 110.9, 67.6, 55.7, 49.2, 45.3, 36.3, 31.6, 29.1, 25.6, 22.5, 14.0; EIMS *m*/*z* (*rel. int*.) 294 (M^+^, 18), 205 (7), 194 (14), 150 (40), 137 (100), 91 (11); HREIMS *m*/*z* 294.1831 [M]^+^ (Calcd for C_17_H_26_O_4_, 294.1831).

**[7]-Gingerol (8c)**: colorless powder (86%), mp 101–102 °C; UV (MeOH) λ_max_ (log ɛ) 281 (3.67), 223 (3.95) nm; IR (KBr) ν_max_ 3524, 2926, 2858, 1705, 1608, 1516, 1369, 1271, 1036, 806 cm^−1; 1^H-NMR (CDCl_3_) δ 6.80 (1H, d, *J =* 8.0 Hz, H-5′), 6.66–6.60 (2H, m, H-2′, -6′), 4.06–3.95 (1H, m, H-5), 3.84 (3H, s, -OCH_3_), 2.86–2.66 (4H, m, H-1, -2), 2.52–2.48 (2H, m, H-4), 1.51–1.25 (10H, m, H-6~10), 0.86 (3H, t, *J =* 6.6 Hz, H-11); ^13^C-NMR (CDCl_3_) δ 211.5, 146.5, 144.0, 132.6, 120.7, 114.5, 111.1, 67.7, 55.8, 49.3, 45.4, 36.5, 31.8, 29.2, 29.1, 25.4, 22.6, 14.1; EIMS *m*/*z* (*rel. int*.) 308 (M^+^, 16), 290 (15), 205 (21), 150 (32), 137 (100), 91 (13), 55 (24); Anal. Calcd. for C_18_H_28_O_4_: C, 70.12%; H, 9.09%; Found: C, 70.14%; H, 9.04%.

**[8]-Gingerol (8d)**: colorless syrup (83%); UV (MeOH) λ_max_ (log ɛ) 282 (3.63), 222 (3.93) nm; IR (neat) ν_max_ 3516, 2928, 2858, 1705, 1608, 1516, 1452, 1271, 1035, 806 cm^−1; 1^H-NMR (CDCl_3_) δ 6.80 (1H, d, *J =* 7.8 Hz, H-5′), 6.66–6.61 (2H, m, H-2′, -6′), 4.05–3.95 (1H, m, H-5), 3.85 (3H, s, -OCH_3_), 2.86–2.67 (4H, m, H-1, -2), 2.53–2.48 (2H, m, H-4), 1.49–1.25 (12H, m, H-6~11), 0.86 (3H, t, *J =* 6.2 Hz, H-12); ^13^C-NMR (CDCl_3_) δ 211.3, 146.4, 143.9, 132.5, 120.6, 114.3, 110.9, 67.6, 55.7, 49.2, 45.3, 36.4, 31.7, 29.4, 29.1, 25.3, 22.5, 14.0; EIMS *m*/*z* (*rel. int*.) 322 (M^+^, 38), 150 (50), 137 (100), 55 (9).

**[9]-Gingerol (8e)**: colorless syrup (84%); UV (MeOH) λ_max_ (log ɛ) 281 (3.33), 224 (3.71) nm; IR (neat) ν_max_ 3535, 2924, 2856, 1704, 1608, 1514, 1369, 1271, 1031, 804 cm^−1; 1^H-NMR (CDCl_3_) δ 6.80 (1H, d, *J =* 7.8 Hz, H-5′), 6.66–6.60 (2H, m, H-2′, -6′), 4.07–3.99 (1H, m, H-5), 3.84 (3H, s, -OCH_3_), 2.86–2.66 (4H, m, H-1, -2), 2.53–2.48 (2H, m, H-4), 1.38–1.25 (14H, m, H-6~12), 0.87 (3H, t, *J =* 6.8 Hz, H-13); ^13^C-NMR (CDCl_3_) δ 211.4, 146.5, 143.9, 132.6, 120.7, 114.5, 111.0, 67.7, 55.8, 49.3, 45.4, 36.5, 31.8, 29.5 (×2), 29.2 (×2), 25.4, 22.6, 14.0; EIMS *m*/*z* (*rel. int*.) 336 (M^+^, 32), 318 (14), 205 (17), 150 (36), 137 (100), 55 (9); HREIMS *m*/*z*: 336.2300 [M]^+^ (Calcd for C_20_H_32_O_4_, 336.2300).

**[10]-Gingerol (8f)**: colorless syrup (85%); UV (MeOH) λ_max_ (log ɛ) 282 (3.43), 224 (3.75) nm; IR (neat) ν_max_ 3439, 2920, 2854, 1706, 1608, 1514, 1369, 1271, 1031, 804 cm^−1; 1^H-NMR (CDCl_3_) δ 6.78 (1H, d, *J =* 7.8 Hz, H-5′), 6.65–6.59 (2H, m, H-2′, -6′), 4.05–3.99 (1H, m, H-5), 3.82 (3H, s, -OCH_3_), 2.83–2.65 (4H, m, H-1, -2), 2.53–2.49 (2H, m, H-4), 1.38–1.25 (16H, m, H-6~13), 0.87 (3H, t, *J =* 6.6 Hz, H-14); ^13^C-NMR (CDCl_3_) δ 211.3, 146.4, 143.8, 132.4, 120.5, 114.4, 110.9, 67.6, 55.6, 49.2, 45.2, 36.3, 31.7, 29.4 (×3), 29.2, 29.1, 25.3, 22.5, 14.0.

#### 3.2.7. General Procedure for the Synthesis of [*n*]-Shogaols (**4a**–**f**)

Conc. HCl (0.1 mL) was added dropwise to a solution of [*n*]-gingerols (**8a**–**f)** (0.54 mmol) in acetone (10 mL) at room temperature. The reaction mixture was stirred for 15 min and then cooled to 0 °C in an ice bath, neutralized by saturated sodium bicarbonate, and extracted with CH_2_Cl_2_ (3 × 10 mL). The organic layers were combined, washed with brine, dried over MgSO_4_, and concentrated under reduced pressure. The crude product was diluted with acetone (10 mL) and then potassium carbonate (0.81 mmol) was added at room temperature. The reaction mixture was stirred for 6h, then cooled to 0 °C in an ice bath, neutralized by 5% HCl_(aq)_, and extracted with CH_2_Cl_2_ (3 × 10 mL). The organic layers were combined, washed with brine, dried over MgSO_4_, and concentrated under reduced pressure. The product was isolated using silica gel column chromatography (ethyl acetate/hexanes = 1/3).

**[5]-Shogaol (4a)**: yellow syrup (86%) [24]; UV (MeOH) λ_max_ (log ɛ) 281 (3.51), 225 (4.31) nm; IR (neat) ν_max_ 3451, 2932, 2862, 1685, 1629, 1514, 1456, 1271, 1034, 984, 806 cm^−1; 1^H-NMR (CDCl_3_) δ 6.89–6.65 (4H, m, H-2′, -5′, -6′, -5), 6.07 (1H, dt, *J =* 15.8, 1.6 Hz, H-4), 5.50 (1H, br s, -OH), 3.87 (3H, s, -OCH_3_), 2.87–2.79 (4H, m, H-1, -2), 2.25–2.14 (2H, m, H-6), 1.49–1.23 (4H, m, H-7, -8), 0.90 (3H, t, *J =* 6.8 Hz, H-9); ^13^C-NMR (CDCl_3_) δ 199.8, 147.8, 146.3, 143.8, 133.2, 130.3, 120.8, 114.3, 111.1, 55.8, 42.0, 32.1, 30.1, 29.9, 22.2, 13.8; EIMS *m*/*z* (*rel. int*.) 262 (M^+^, 46), 205 (42), 151 (16), 137 (100), 55 (22).

**[6]-Shogaol (4b)**: yellow syrup (85%) [24]; UV (MeOH) λ_max_ (log ɛ) 282 (3.47), 224 (4.25) nm; IR (neat) ν_max_ 3424, 2928, 2860, 1662, 1616, 1514, 1456, 1271, 1034, 982, 808 cm^−1; 1^H-NMR (CDCl_3_) δ 6.89–6.65 (4H, m, H-2′, -5′, -6′, -5), 6.08 (1H, dt, *J =* 16.0, 1.4 Hz, H-4), 5.54 (1H, br s, -OH), 3.86 (3H, s, -OCH_3_), 2.89–2.79 (4H, m, H-1, -2), 2.24–2.13 (2H, m, H-6), 1.51–1.26 (6H, m, H-7~9), 0.88 (3H, t, *J =* 6.5 Hz, H-10); ^13^C-NMR (CDCl_3_) δ 199.8, 147.8, 146.3, 143.8, 133.2, 130.2, 120.7, 114.2, 111.0, 55.8, 41.9, 32.4, 31.3, 29.8, 27.1, 22.4, 13.9; EIMS *m*/*z* (*rel. int*.) 276 (M^+^, 43), 205 (52), 151 (16), 137 (100), 119 (10), 55 (18).

**[7]-Shogaol (4c)**: yellow syrup (83%) [24]; UV (MeOH) λ_max_ (log ɛ) 282 (3.44), 225 (4.18) nm; IR (neat) ν_max_ 3450, 2927, 2856, 1691, 1626, 1516, 1460, 1367, 1271, 1036, 978, 815 cm^−1; 1^H-NMR (CDCl_3_) δ 6.89–6.65 (4H, m, H-2′, -5′, -6′, -5), 6.08 (1H, dt, *J =* 16.0, 1.5 Hz, H-4), 3.86 (3H, s, -OCH_3_), 2.86–2.82 (4H, m, H-1, -2), 2.24–2.14 (2H, m, H-6), 1.47–1.26 (8H, m, H-7~10), 0.88 (3H, t, *J =* 6.7 Hz, H-11); ^13^C-NMR (CDCl_3_) δ 199.8, 147.9, 146.4, 143.8, 133.2, 130.3, 120.8, 114.3, 111.1, 55.8, 42.0, 32.5, 31.5, 29.8, 28.8, 28.0, 22.5, 14.0; EIMS *m*/*z* (*rel. int*.) 290 (M^+^, 27), 205 (28), 151 (14), 137 (100), 55 (9).

**[8]-Shogaol (4d)**: yellow syrup (79%) [24]; UV (MeOH) λ_max_ (log ɛ) 282 (3.72), 225 (4.52) nm; IR (neat) ν_max_ 3433, 2926, 2856, 1675, 1629, 1514, 1456, 1271, 1034, 980, 808 cm^−1; 1^H-NMR (CDCl_3_) δ 6.89–6.65 (4H, m, H-2′, -5′, -6′, -5), 6.08 (1H, dt, *J =* 15.8, 1.6 Hz, H-4), 3.86 (3H, s, -OCH_3_), 2.85–2.82 (4H, m, H-1, -2), 2.24–2.13 (2H, m, H-6), 1.47–1.27 (10H, m, H-7~11), 0.88 (3H, t, *J =* 6.7 Hz, H-12); ^13^C-NMR (CDCl_3_) δ 199.8, 147.9, 146.4, 143.8, 133.2, 130.2, 120.7, 114.3, 111.1, 55.8, 41.9, 32.4, 31.7, 29.8, 29.1, 28.0, 22.6, 14.0; EIMS *m*/*z* (*rel. int*.) 304 (M^+^, 34), 205 (51), 151 (18), 137 (100), 69 (20), 55 (26).

**[9]-Shogaol (4e)**: yellow syrup (85%) [24]; UV (MeOH) λ_max_ (log ɛ) 282 (3.55), 226 (4.36) nm; IR (neat) ν_max_ 3432, 2926, 2856, 1685, 1638, 1514, 1471, 1271, 1034, 982, 808 cm^−1; 1^H-NMR (CDCl_3_) δ 6.91–6.66 (4H, m, H-2′, -5′, -6′, -5), 6.08 (1H, dt, *J =* 15.7, 1.4 Hz, H-4), 3.87 (3H, s, -OCH_3_), 2.87–2.80 (4H, m, H-1, -2), 2.25–2.14 (2H, m, H-6), 1.47–1.27 (12H, m, H-7~12), 0.88 (3H, t, *J =* 6.6 Hz, H-13); ^13^C-NMR (CDCl_3_) δ 199.8, 147.9, 146.4, 143.9, 133.2, 130.3, 120.8, 114.3, 111.1, 55.9, 42.0, 32.5, 31.8, 29.9, 29.3, 29.1, 28.1, 22.6, 14.1; EIMS *m*/*z* (*rel. int*.) 318 (M^+^, 35), 205 (58), 151 (16), 137 (100), 55 (15).

**[10]-Shogaol (4f)**: yellow syrup (88%) [24]; UV (MeOH) λ_max_ (log ɛ) 284 (3.45), 225 (4.16) nm; IR (neat) ν_max_ 3513, 2924, 2852, 1688, 1638, 1512, 1471, 1271, 1034, 982, 808 cm^−1; 1^H-NMR (CDCl_3_) δ 6.89–6.64 (4H, m, H-2′, -5′, -6′, -5), 6.08 (1H, dt, *J =* 15.6, 1.4 Hz, H-4), 3.85 (3H, s, -OCH_3_), 2.84–2.82 (4H, m, H-1, -2), 2.19–2.13 (2H, m, H-6), 1.43–1.25 (14H, m, H-7~13), 0.87 (3H, t, *J =* 6.4 Hz, H-14); ^13^C-NMR (CDCl_3_) δ 199.9, 147.9, 146.4, 143.9, 133.2, 130.3, 120.7, 114.3, 111.1, 55.8, 41.9, 32.5, 31.8, 29.8, 29.4, 29.3, 29.2, 29.1, 28.1, 22.6, 14.1.

#### 3.2.8. General Procedure for the Synthesis of [*n*]-Isodehydrogingerdiones (**1a**–**f**)

DMSO (0.15 mL, 2.16 mmol) was added dropwise to a solution of oxalyl chloride (0.12 mL, 1.40 mmol) in acetone (10 mL) at −50–60 °C under argon. The reaction mixture was stirred for 3 min, and then a solution of [*n*]-dehydrogingerols (**3a**–**f**) (1.08 mmol) in CH_2_Cl_2_ (5 mL) was slowly added. The reaction mixture was stirred for another 15 min, Et_3_N was added to the mixture; the temperature was changed to 0 °C in an ice bath for 20 min, and the reaction mixture was then neutralized using 5% HCl_(aq)_ and extracted with CH_2_Cl_2_ (3 × 10 mL). The organic layers were combined, washed with brine, dried over MgSO_4_, and concentrated under reduced pressure. The product [*n*]-isodehydrogingerdiones (**1a**–**f**) and [*n*]-dehydroshogaols (**2a**–**f**) were isolated using silica gel column chromatography (ethyl acetate/hexanes = 1/3).

**[5]-Isodehydrogingerdione (1a)**: yellow syrup (51%); UV (MeOH) λ_max_ (log ɛ) 369 (4.39), 255 (3.71) nm; IR (KBr) ν_max_ 3358, 2958, 2866, 1634, 1576, 1512, 1427, 1273, 1030, 966, 837 cm^−1; 1^H-NMR (CDCl_3_) δ 7.51 (1H, d, *J =* 15.8 Hz, H-1), 7.07 (1H, dd, *J =* 8.2, 1.8 Hz, H-6′), 7.00 (1H, d, *J =* 1.8 Hz, H-2′), 6.90 (1H, d, *J =* 8.2 Hz, H-5′), 6.34 (1H, d, *J =* 15.8 Hz, H-2), 5.62 (1H, s, H-4), 3.91 (3H, s, -OCH_3_), 2.38 (2H, t, *J =* 7.2 Hz, H-6), 1.70–1.58 (2H, m, H-7), 1.46–1.28 (2H, m, H-8), 0.92 (3H, t, *J =* 7.2 Hz, H-9); ^13^C-NMR (CDCl_3_) δ 200.2, 178.0, 147.6, 146.8, 139.8, 127.6, 122.6, 120.5, 114.8, 109.4, 100.1, 55.9, 39.8, 27.7, 22.4, 13.8.

**[6]-Isodehydrogingerdione (1b)**: yellow syrup (49%); UV (MeOH) λ_max_ (log ɛ) 369 (4.37), 256 (3.70) nm; IR (KBr) ν_max_ 3418, 2956, 2864, 1634, 1591, 1512, 1427, 1271, 1032, 970, 816 cm^−1; 1^H-NMR (CDCl_3_) δ 7.51 (1H, d, *J =* 15.8 Hz, H-1), 7.06 (1H, dd, *J =* 8.0, 1.8 Hz, H-6′), 7.01 (1H, d, *J =* 1.8 Hz, H-2′), 6.90 (1H, d, *J =* 8.0 Hz, H-5′), 6.34 (1H, d, *J =* 15.8 Hz, H-2), 5.61 (1H, s, H-4), 3.94 (3H, s, -OCH_3_), 2.37 (2H, t, *J =* 7.4 Hz, H-6), 1.69–1.57 (2H, m, H-7), 1.35–1.28 (2H, m, H-8~9), 0.90 (3H, t, *J =* 6.2 Hz, H-10); ^13^C-NMR (CDCl_3_) δ 200.2, 178.0, 147.6, 146.7, 139.8, 127.7, 122.6, 120.5, 114.8, 109.4, 100.1, 55.9, 40.1, 31.4, 25.3, 22.4, 13.9.

**[7]-Isodehydrogingerdione (1c)**: yellow syrup (59%); UV (MeOH) λ_max_ (log ɛ) 368 (4.17), 257 (3.62) nm; IR (KBr) ν_max_ 3423, 2928, 2858, 1634, 1582, 1512, 1427, 1273, 1031, 974, 814 cm^−1; 1^H-NMR (CDCl_3_) δ 7.52 (1H, d, *J =* 15.8 Hz, H-1), 7.08 (1H, dd, *J =* 8.2, 1.8 Hz, H-6′), 7.02 (1H, d, *J =* 1.8 Hz, H-2′), 6.91 (1H, d, *J =* 8.2 Hz, H-5′), 6.33 (1H, d, *J =* 15.8 Hz, H-2), 5.94 (1H, br s, -OH), 5.62 (1H, s, H-4), 3.93 (3H, s, -OCH_3_), 2.37 (2H, t, *J =* 7.6 Hz, H-6), 1.64–1.61 (2H, m, H-7), 1.30–1.24 (6H, m, H-8~10), 0.89 (3H, t, *J =* 6.6 Hz, H-11); ^13^C-NMR (CDCl_3_) δ 200.2, 178.0, 147.7, 146.8, 139.8, 127.7, 122.6, 120.5, 114.8, 109.5, 100.1, 55.9, 40.1, 31.6, 29.0, 25.6, 22.5, 14.0.

**[9]-Isodehydrogingerdione (1e)**: yellow syrup (48%); UV (MeOH) λ_max_ (log ɛ) 369 (4.37), 254 (3.72) nm; IR (KBr) ν_max_ 3423, 2955, 2854, 1634, 1583, 1512, 1427, 1271, 1031, 970, 816 cm^−1; 1^H-NMR (CDCl_3_) δ 7.52 (1H, d, *J =* 15.6 Hz, H-1), 7.08 (1H, dd, *J =* 8.2, 1.8 Hz, H-6′), 7.01 (1H, d, *J =* 1.8 Hz, H-2′), 6.91 (1H, d, *J =* 8.2 Hz, H-5′), 6.34 (1H, d, *J =* 15.6 Hz, H-2), 5.91 (1H, br s, -OH), 5.62 (1H, s, H-4), 3.93 (3H, s, -OCH_3_), 2.37 (2H, t, *J =* 7.2 Hz, H-6), 1.67–1.60 (2H, m, H-7), 1.29–1.27 (10H, m, H-8~12), 0.88 (3H, t, *J =* 6.2 Hz, H-13); ^13^C-NMR (CDCl_3_) δ 200.1, 177.9, 147.6, 146.7, 140.0, 127.6, 122.5, 120.5, 114.7, 109.4, 100.1, 55.9, 40.1, 31.8, 29.3, 29.1, 25.6, 22.6, 14.0.

### 3.3. Antiplatelet Aggregatory Bioassay

An assay of the antiplatelet aggregatory activity of the isolated compound was conducted according to the procedures of Teng and coworkers [25,26]. Washed platelets were prepared from blood withdrawn with a siliconized syringe from the marginal vein of New Zealand rabbits. The platelet suspension was obtained from EDTA-anticoagulated platelet-rich plasma according to the washing procedure described previously. The platelet number was determined using a cell counter (Hema-laser 2, Sebia, France) and adjusted to 3.0 × 10^8^ platelets/mL. The platelet pellets were suspended in Tyrode’s solution containing Ca^2+^ (1 mM) and bovine serum albumin (0.35%). All glassware was siliconized. Platelet aggregation was measured using the turbidimetric method [26]. The aggregations were measured with a Lumi-aggregometer (Model 1020, Payton, Canada) connected to two dual-channel recorders.

## 4. Conclusions

Eight groups of derivatives based on the skeletons of shogaol and gingerol, the active pungent principles from ginger, were synthesized and evaluated for their antiplatelet bioactivity. Among the compounds synthesized, [6]-paradol **5b** displayed the most significant inhibition of platelet aggregation induced by AA. Anti-PAF induced platelet aggregation activity was not found in the present study, suggesting that [6]-paradol **5b** is a selective inhibitor. The traditional use of *Z. officinale* is to promote the blood circulation necessary for removing blood stasis, and the results of this study substantiated the anti-platelet aggregation activity of these synthetic derivatives related to shogaol and gingerol. It is valuable to explore new anti-platelet aggregation drugs based on the skeleton of [*n*]-paradol or other principles reported from the *Zingiber* series.

## Figures and Tables

**Table 1.** The yields of products **2** and **3** from cross aldol condensation.

[*n*]	2 yield%	3 yield%
**a**: *n =* 5	9	65
**b**: *n =* 6	15	59
**c**: *n =* 7	13	56
**d**: *n =* 8	15	66
**e**: *n =* 9	13	58
**f**: *n =* 10	6	50

**Table 2.** Cross aldol condensation under air atmosphere.

[*n*]	2 yield%	7 yield%
**a**: *n =* 5	19	8
**b**: *n =* 6	21	10
**c**: *n =* 7	18	9
**d**: *n =* 8	18	9
**e**: *n =* 9	16	8
**f**: *n =* 10	15	8

**Table 3.** Hydrogenation of compound **2** to afford product **5** and **6**.

[*n*]	5 yield%	6 yield%
**a**: *n =* 5	79	80
**b**: *n =* 6	78	76
**c**: *n =* 7	81	75
**d**: *n =* 8	77	73
**e**: *n =* 9	80	74
**f**: *n =* 10	79	79

**Table 4.** Hydrogenation and elimination of compound **3** to yield products **8** and **4**.

[*n*]	8 yield%	4 yield%
**a**: *n =* 5	85	86
**b**: *n =* 6	84	85
**c**: *n =* 7	86	83
**d**: *n =* 8	83	79
**e**: *n =* 9	84	85
**f**: *n =* 10	85	88

**Table 5.** The Swern oxidation of compound **3** to obtain products **2** and **1**.

[*n*]	2 yield%	1 yield%
**a**: *n =* 5	16	51
**b**: *n =* 6	15	49
**c**: *n =* 7	11	59
**e**: *n =* 9	17	48

**Table 6.** Antiplatelet activity of [*n*]-paradols **5a**–**f**.

Inhibition (%)								

Inducer	Control	Conc. (μg/mL)	5a	5b	5c	5d	5e	5f
AA (100 μM)	0.0 ± 1.3	10	100.0 ± 1.3 ***	100.0 ± 1.3 ***	100.0 ± 1.3 ***	100.0 ± 1.3 ***	100.0 ± 0.4	100.0 ± 0.4 ***
		5	-	-	100.0 ± 1.3 ***	100.0 ± 1.3 ***	-	100.0 ± 0.4 ***
		2	-	100.0 ± 1.3 ***	94.9 ± 3.4 ***	98.4 ± 0.2 ***	100.0 ± 0.4 ***	87.4 ± 4.8 ***
		1	100.0 ± 1.3 ***	97.0 ± 1.4 ***	93.2 ± 5.0 ***	90.9 ± 4.0 ***	96.9 ± 2.1 ***	75.9 ± 4.7 ***
		0.5	87.1 ± 6.5 ***	92.8 ± 5.4 ***	90.0 ± 7.9 ***	81.6 ± 9.4 ***	89.8 ± 3.8 ***	53.6 ± 2.1 ***
		0.2	63.9 ± 10.6 ***	79.9 ± 8.9 ***	76.0 ± 10.7 ***	76.9 ± 12.1 ***	21.7 ± 3.4 ***	26.4 ± 6.1 ***
		0.1	57.3 ± 12.9 ***	67.6 ± 13.0 ***	68.0 ± 1.7 ***	53.0 ± 13.7 **	-	6.4 ± 2.2 **
		0.05	29.0 ± 13.6	52.6 ± 13.1 ***	17.0 ± 5.0 *	26.7 ± 8.9 *	-	-
		0.02	10.0 ± 3.5 *	14.7 ± 5.6	7.0 ± 3.9	8.1 ± 2.9	-	-
		0.01	−0.9 ± 0.0	5.5 ± 1.4	2.4 ± 1.7	-	-	-
*IC*_50_ (μg/mL)	-	-	0.10	0.07	0.12	0.12	0.30	0.49
Col (10 μM)	0.0 ± 0.8	10	61.7 ± 3.8 ***	33.3 ± 4.1 ***	39.7 ± 3.8 ***	52.7 ± 3.5 ***	36.9 ± 5.4 ***	32.9 ± 3.1 ***
PAF (2 ng/mL)	0.0 ± 1.0	10	1.9 ± 0.8 ***	1.9 ± 0.3	0.7 ± 0.1	1.3 ± 0.0	−0.8 ± 0.4	21 ± 1.6
Thr (0.1 U/mL)	0.0 ± 1.0	10	−2.1 ± 0.2 *	0.0 ± 0.6	−2.7 ± 0.1	−1.0 ± 0.2	0.2 ± 0.4	2.5 ± 0.4

* *p <* 0.05;

** *p <* 0.01;

*** *p <* 0.001.

**Table 7.** Antiplatelet activity of [*n*]-dehydroparadols **6a**–**f**.

Inhibition (%)								

Inducer	Control	Conc. (μg/mL)	6a	6b	6c	6d	6e	6f
AA (100 μM)	0.0 ± 0.4	10	100.0 ± 0.4 ***	100.0 ± 0.4	100.0 ± 0.4 ***	100.0 ± 0.4 ***	100.0 ± 0.4 ***	100.0 ± 0.4 ***
		5	-	100.0 ± 0.4	97.7 ± 1.6 ***	98.4 ± 0.9 ***	-	-
		2	100.0 ± 0.4 ***	94.9 ± 4.0	96.6 ± 2.5 ***	93.1 ± 5.1 ***	100.0 ± 0.4 ***	100.0 ± 0.4 ***
		1	78.4 ± 8.5 ***	64.9 ± 10.7	86.3 ± 7.4 ***	73.1 ± 1.0 ***	75.2 ± 7.8 ***	97.7 ± 1.6 ***
		0.5	64.2 ± 9.1 ***	50.4 ± 7.2	59.4 ± 7.0 ***	62.3 ± 0.7 ***	55.2 ± 11.6 ***	97.7 ± 1.6 ***
		0.2	38.9 ± 10.8 **	14.5 ± 4.8	40.8 ± 4.0 ***	36.3 ± 0.0 ***	8.7 ± 1.0 ***	79.4 ± 7.7 ***
		0.1	14.4 ± 6.5	6.7 ± 3.2	16.1 ± 1.9 ***	12.2 ± 3.1 *	-	31.4 ± 8.6 *
		0.05	3.4 ± 0.9	-	6.7 ± 1.3 *	-	-	5.4 ± 1.9 *
*IC*_50_ (μg/mL)	-	-	0.32	0.56	0.35	0.40	0.52	0.16
Col (10 μM)	0.0 ± 1.0	10	42.8 ± 4.7 ***	39.3 ± 4.6 ***	18.4 ± 1.6 ***	36.1 ± 0.1 *	20.2 ± 2.6 ***	18.9 ± 3.4 ***
PAF (2 ng/mL)	0.0 ± 1.0	10	5.3 ± 3.3	0.4 ± 0.2	0.5 ± 0.5	2.0 ± 2.5	1.1 ± 0.3	−1.0 ± 0.5
Thr (0.1 U/mL)	0.0 ± 0.9	10	4.3 ± 0.1 **	1.8 ± 0.6	3.0 ± 1.6	1.5 ± 0.5	5.0 ± 0.1	0.9 ± 0.5

* *p <* 0.05;

** *p <* 0.01;

*** *p <* 0.001.

**Table 8.** Antiplatelet activity of [*n*]-shogaols **4a**–**f**.

Inhibition (%)								

Inducer	Control	Conc. (μg/mL)	4a	4b	4c	4d	4e	4f
AA (100 μM)	0.0 ± 1.2	10	100.0 ± 1.2 ***	100.0 ± 1.2 ***	100.0 ± 1.2 ***	100.0 ± 1.2 ***	100.0 ± 1.2 ***	100.0 ± 1.2 ***
		1	100.0 ± 1.2 ***	100.0 ± 1.2 ***	-	-	-	100.0 ± 1.2 ***
		0.5	70.0 ± 19.6 ***	94.2 ± 3.4 ***	100.0 ± 1.2 ***	100.0 ± 1.2 ***	100.0 ± 1.2 ***	83.1 ± 6.0 ***
		0.2	52.8 ± 22.1 **	78.0 ± 15.2 ***	62.3 ± 18.1 **	74.9 ± 14.3 ***	57.9 ± 1.1 ***	81.2 ± 9.1 ***
		0.1	14.7 ± 8.3	47.3 ± 19.6 *	54.1 ± 21.7 *	14.3 ± 16.0 **	36.0 ± 14.4 *	41.8 ± 17.8 *
		0.05	5.3 ± 2.7	13.7 ± 5.4 *	7.9 ± 2.7	5.2 ± 2.2	7.2 ± 2.7	30.9 ± 21.5
		0.02	-	2.8 ± 1.1	-	-	-	2.8 ± 0.2

*IC*_50_ (μg/mL)	-	-	0.23	0.12	0.13	0.15	0.15	0.11

Col (10 μM)	0.0 ± 1.7	10	91.2 ± 6.0 ***	84.5 ± 11.9 ***	78.7 ± 13.2 ***	74.5 ± 14.0 ***	79.3 ± 16.2 ***	91.1 ± 1.9 ***
		5	55.3 ± 7.7 ***	50.1 ± 5.4 ***	41.4 ± 4.3 ***	38.0 ± 7.9 ***	45.5 ± 14.7 **	82.8 ± 7.3 ***
		2	20.0 ± 6.6 *	13.3 ± 2.5 **	9.8 ± 0.6 **	6.0 ± 1.2	27.0 ± 15.6	36.3 ± 14.9 *
		1	1.5 ± 0.7	2.5 ± 2.5	1.7 ± 0.8	-	4.0 ± 2.0	11.3 ± 3.3 *
		0.5	-	-	-	-	-	3.2 ± 0.7

PAF (2 ng/mL)	0.0 ± 1.2	10	9.3 ± 0.1 ***	6.4 ± 0.7 *	6.0 ± 1.3	5.2 ± 1.1	4.5 ± 2.1	6.6 ± 2.2

Thr (0.1 U/mL)	0.0 ± 0.4	10	3.5 ± 0.6 *	1.7 ± 0.2	1.5 ± 0.2	2.2 ± 0.6	1.0 ± 1.0	2.2 ± 3.4

* *p <* 0.05;

** *p <* 0.01;

*** *p <* 0.001.

**Table 9.** Antiplatelet activity of [*n*]-dehydroshogaols **2a**–**f**.

Inhibition (%)								

Inducer	Control	Conc. (μg/mL)	2a	2b	2c	2d	2e	2f
AA (100 μM)	0.0 ± 1.4	10	100.0 ± 1.4 ***	100.0 ± 1.4 ***	100.0 ± 1.4 ***	100.0 ± 1.4 ***	100.0 ± 1.4 ***	100.0 ± 1.4 ***
		5	100.0 ± 1.4 ***	100.0 ± 1.4 ***	100.0 ± 1.4 ***	100.0 ± 1.4 ***	100.0 ± 1.4 ***	96.9 ± 1.1 ***
		2	86.4 ± 7.0 ***	71.1 ± 13.0 ***	84.1 ± 12.4 ***	86.4 ± 9.7 ***	97.7 ± 0.6 ***	95.5 ± 2.3 ***
		1	35.6 ± 12.4 ***	30.7 ± 8.6 **	61.4 ± 18.1 **	54.5 ± 13.4 ***	91.1 ± 6.4 ***	81.4 ± 7.7 ***
		0.5	26.5 ± 18.4	19.7 ± 12.9	19.2 ± 8.1 *	9.2 ± 0.3 ***	31.7 ± 18.3	60.5 ± 19.6 **
		0.2	7.2 ± 3.5	3.3 ± 0.5	5.6 ± 0.9 *	1.5 ± 0.5	8.6 ± 4.2	3.3 ± 1.5
		0.1	-	-	-	-	2.1 ± 0.9	-

*IC*_50_ (μg/mL)	-	-	0.96	1.18	0.88	0.99	0.57	0.51

Col (10 μM)	0.0 ± 0.6	10	93.3 ± 5.3 ***	26.9 ± 9.2 *	66.8 ± 14.1 ***	51.6 ± 14.8 **	44.8 ± 7.8 ***	62.1 ± 13.9 ***
		5	29.1 ± 8.0 **	2.1 ± 0.9	7.1 ± 1.5 **	8.3 ± 1.3 ***	-	-
		2	3.5 ± 1.1	-	-	-	-	-
		0.2	92.2 ± 2.2 ***	-	-	-	-	-

PAF (2 ng/mL)	0.0 ± 1.3	10	11.2 ± 1.4 **	5.0 ± 3.1	6.8 ± 0.7 *	5.9 ± 0.7 *	5.9 ± 1.0 *	5.6 ± 0.7 *

Thr (0.1 U/mL)	0.0 ± 0.4	10	5.1 ± 2.1	1.6 ± 1.4	0.9 ± 1.3	1.8 ± 1.6	3.2 ± 1.2	1.0 ± 1.3

* *p <* 0.05;

** *p <* 0.01;

*** *p <* 0.001.

**Table 10.** Antiplatelet activity of [*n*]-gingerols **8a**–**f**.

Inhibition (%)								

Inducer	Control	Conc. (μg/mL)	8a	8b	8c	8d	8e	8f
AA (100 μM)	0.0 ± 1.3	10	93.0 ± 2.6 ***	100.0 ± 1.3 ***	93.8 ± 4.2 ***	96.3 ± 1.1 ***	100.0 ± 1.3 ***	100.0 ± 1.3 ***
		5	59.9 ± 13.4 ***	100.0 ± 1.3 ***	79.6 ± 10.5 ***	90.4 ± 4.3 ***	97.0 ± 1.4 ***	99.2 ± 0.5 ***
		2	37.4 ± 15.2 *	75.6 ± 9.7 ***	74.8 ± 12.9 ***	84.1 ± 8.1 ***	77.1 ± 12.4 ***	89.2 ± 5.9 ***
		1	22.7 ± 12.6	38.7 ± 4.7 ***	63.1 ± 16.7 ***	81.7 ± 9.9 ***	72.1 ± 13.4 ***	80.5 ± 9.9 ***
		0.5	6.6 ± 3.5	22.3 ± 3.1 ***	47.3 ± 18.4 *	71.3 ± 14.7 ***	59.4 ± 15.0 **	67.8 ± 10.5 ***
		0.2	-	7.0 ± 0.7 *	27.7 ± 16.7	64.2 ± 15.4 ***	3.8 ± 1.3	20.9 ± 6.8
		0.1	-	-	4.8 ± 3.1	34.3 ± 14.2 *	-	12.8 ± 4.1 *
		0.05	-	-	2.1 ± 2.3	8.0 ± 2.2	-	1.8 ± 0.8
*IC*_50_ (μg/mL)	-	-	2.72	1.04	0.72	0.23	0.65	0.45
Col (10 μM)	0.0 ± 0.8	10	20.1 ± 2.1 ***	28.3 ± 6.3 **	41.6 ± 4.7 ***	-	6.6 ± 1.9 *	49.3 ± 6.3 ***
PAF (2 ng/mL)	0.0 ± 1.0	10	−0.6 ± 0.4	0.4 ± 0.0	0.2 ± 0.2	-	−0.4 ± 0.6	−0.3 ± 0.3
Thr (0.1 U/mL)	0.0 ± 1.4	10	−0.5 ± 0.2	−2.7 ± 1.2	−1.2 ± 0.3	0.7 ± 0.1	−1.6 ± 0.5	−4.3 ± 0.1

* *p <* 0.05;

** *p <* 0.01;

*** *p <* 0.001.

**Table 11.** Antiplatelet activity of [*n*]-dehydrogingerols **3a**–**f**.

Inhibition (%)								

Inducer	Control	Conc. (μg/mL)	3a	3b	3c	3d	3e	3f
AA (100 μM)	0.0 ± 0.8	10	78.0 ± 12.7 ***	98.8 ± 0.2 ***	100.0 ± 0.8 ***	84.7 ± 12.4 ***	92.9 ± 5.0 ***	100.0 ± 0.8 ***
		5	63.0 ± 14.3 ***	53.7 ± 11.1 **	76.2 ± 12.0 ***	65.5 ± 16.5 ***	83.2 ± 12.9 ***	100.0 ± 0.8 ***
		2	30.4 ± 19.4	25.7 ± 13.8	15.3 ± 3.9 ***	52.4 ± 21.7 *	19.6 ± 3.9 ***	100.0 ± 0.8 ***
		1	8.5 ± 6.9	16.4 ± 13.5	7.0 ± 3.5	4.4 ± 1.0 *	5.7 ± 0.2 ***	9.8 ± 3.0 **
		0.5	2.8 ± 1.7	6.5 ± 4.8	-	-	-	2.6 ± 0.9
*IC*_50_ (μg/mL)	-	-	3.59	4.74	3.19	2.99	3.14	1.20
Col (10 μM)	0.0 ± 0.6	10	7.5 ± 4.2	16.9 ± 8.4	8.7 ± 3.7	10.6 ± 3.3	40.0 ± 7.1 ***	41.3 ± 14.2 *
PAF (2 ng/mL)	0.0 ± 1.3	10	3.1 ± 1.0	4.3 ± 0.8	1.4 ± 0.2	1.5 ± 0.8	3.0 ± 1.8	−0.9 ± 0.6
Thr (0.1 U/mL)	0.0 ± 0.4	10	1.9 ± 0.3	1.7 ± 1.0	0.7 ± 0.6	0.4 ± 0.6	1.0 ± 0.4	1.7 ± 0.1

* *p <* 0.05;

** *p <* 0.01;

*** *p <* 0.001.

**Table 12.** Antiplatelet activity of [*n*]-isodehydrogingerdiones **1a**–**c** and **e**.

Inhibition (%)						

Inducer	Control	Conc. (μg/mL)	1a	1b	1c	1e
AA (100 μM)	0.0 ± 0.4	10	100.0 ± 0.4 ***	100.0 ± 0.4 ***	98.4 ± 0.9 ***	100.0 ± 0.4 ***
		5	100.0 ± 0.4 ***	100.0 ± 0.4 ***	-	100.0 ± 0.4 ***
		2	94.3 ± 4.2 ***	93.1 ± 5.5 ***	100.0 ± 0.4 ***	82.7 ± 6.0 ***
		1	30.8 ± 6.4	59.6 ± 6.0 ***	53.4 ± 4.2 ***	74.7 ± 7.8 ***
		0.5	10.6 ± 2.9 **	7.4 ± 4.1	40.3 ± 9.9 **	26.0 ± 5.4 ***
		0.2	-	-	3.6 ± 1.6	10.2 ± 4.4
*IC*_50_ (μg/mL)	-	-	1.28	1.03	0.68	0.75
Col (10 μM)	0.0 ± 1.0	10	53.3 ± 6.3 ***	38.9 ± 10.0 ***	35.2 ± 6.6 ***	36.6 ± 13.8 *
PAF (2 ng/mL)	0.0 ± 1.0	10	0.1 ± 0.4	0.2 ± 0.4	0.2 ± 0.4	2.3 ± 0.3
Thr (0.1 U/mL)	0.0 ± 0.9	10	1.7 ± 0.2	1.7 ± 0.4	0.2 ± 0.1	1.1 ± 0.2

* *p <* 0.05;

** *p <* 0.01;

*** *p <* 0.001.

**Table 13.** Antiplatelet activity of [*n*]-epoxydehydroparadols **7a**–**f**.

Inhibition (%)								

Inducer	Control	Conc. (μg/mL)	7a	7b	7c	7d	7e	7f
AA (100 μM)	0.0 ± 0.8	10	96.5 ± 2.3	100.0 ± 0.8	100.0 ± 0.8 ***	100.0 ± 0.8 ***	73.8 ± 13.6 ***	100.0 ± 0.8 ***
		5	87.4 ± 8.5	93.8 ± 4.5	95.7 ± 3.0 ***	67.8 ± 16.5 ***	-	96.5 ± 2.3 ***
		2	46.1 ± 13.7	77.9 ± 10.7	80.1 ± 16.3 ***	51.1 ± 20.2 **	-	83.7 ± 10.7 ***
		1	12.6 ± 3.2	51.1 ± 14.6	41.5 ± 12.1 ***	22.5 ± 14.1 *	-	44.5 ± 18.6 ***
		0.5	-	9.4 ± 2.5	25.1 ± 11.0 **	5.1 ± 0.0 ***	-	37.0 ± 17.6 ***
		0.2	-	-	4.6 ± 0.8 **	-	-	4.1 ± 0.3 ***
*IC*_50_ (μg/mL)	-	-	2.38	1.24	1.09	2.24	-	0.96
Col (10 μM)	0.0 ± 1.9	10	45.2 ± 14.0 *	1.8 ± 1.4 **	20.2 ± 7.7	10.7 ± 3.6	3.1 ± 1.0	35.7 ± 0.9 ***
PAF (2 ng/mL)	0.0 ± 0.7	10	4.4 ± 1.7	4.3 ± 0.8 *	2.8 ± 0.8	4.7 ± 0.1 ***	1.3 ± 0.1	1.9 ± 0.3
Thr (0.1 U/mL)	0.0 ± 0.3	10	1.7 ± 1.1	−0.2 ± 0.1	−0.2 ± 0.7	−0.2 ± 0.8	−1.1 ± 1.2	0.0 ± 0.4

* *p <* 0.05;

** *p <* 0.01;

*** *p <* 0.001.
